# Redefining the differences in gene content between *Yersinia pestis* and *Yersinia pseudotuberculosis* using large-scale comparative genomics

**DOI:** 10.1099/mgen.0.000028

**Published:** 2015-08-03

**Authors:** Katy J. Califf, Paul S. Keim, David M. Wagner, Jason W. Sahl

**Affiliations:** ^1^​ Microbial Center for Microbial Genetics and Genomics, Northern Arizona University, Flagstaff, AZ, USA; ^2^​ Translational Genomics Research Institute, Flagstaff, AZ, USA

**Keywords:** *Yersinia pestis*, genomics, comparative genomics, evolution

## Abstract

*Yersinia pestis*, the causative agent of plague, is best known for historical pandemics, but still actively causes disease in many parts of the world. *Y. pestis* is a recently derived clone of the pathogenic species *Yersinia pseudotuberculosis*, but is more associated with human infection. Numerous studies have documented genomic changes since the two species differentiated, although all of these studies used a relatively small sample set for defining these differences. In this study, we compared the complete genomic content between a diverse set of *Y. pestis* and *Y. pseudotuberculosis* genomes, and identified unique loci that could serve as diagnostic markers or for better understanding the evolution and pathogenesis of each group. Comparative genomics analyses also identified subtle variations in gene content between individual monophyletic clades within these species, based on a core genome single nucleotide polymorphism phylogeny that would have been undetected in a less comprehensive genome dataset. We also screened loci that were identified in other published studies as unique to either species and generally found a non-uniform distribution, suggesting that the assignment of these unique genes to either species should be re-evaluated in the context of current sequencing efforts. Overall, this study provides a high-resolution view into the genomic differences between *Y. pestis* and *Y. pseudotuberculosis*, demonstrating fine-scale differentiation and unique gene composition in both species.

## Data Summary

1. *De novo* sequencing data of 118 *Yersinia pestis* strains are deposited in the Sequence Read Archive: http://www.ncbi.nlm.nih.gov/Traces/sra/?study = SRP003808

## Impact Statement

This study employed large-scale comparative genomics to understand the genomic differences between *Yersinia pseudotuberculosis* and *Yersinia pestis*, a pathogen responsible for global human pandemics. We identified that many results from previous comparative studies were not reproduced when using a larger sample set. These results change the understanding of the evolution of *Y. pestis*, specifically the timing for when genomic components were gained and lost since the most recent common ancestor between the two species. Additionally, the identification of coding regions conserved in *Y. pestis* and missing from *Y. pseudotuberculosis* provides targets for functional studies to better understand the pathogenicity and spread of *Y. pestis*. Whilst the results of this study will primarily appeal to researchers who study *Yersinia* spp., the methods employed provide a framework to better understand the evolution of the pan-genome in any bacterial species.

## Introduction

*Yersinia pestis*, the causative agent of plague, is a recently evolved clone of *Yersinia pseudotuberculosis* serotype O : 1b (Achtman *et al.*, 1999). *Y. pestis* is best known for causing three pandemics, including the black death (Achtman *et al.*, 2004), the plague of Justinian (Wagner *et al.*, 2014) and the third pandemic, where *Y. pestis* was spread worldwide (Cui *et al.*, 2013). *Y. pestis* still actively causes disease in many parts of the world, including North America (Wagner *et al.*, 2010) and Madagascar (Ratovonjato *et al.*, 2014). Disease caused by *Y. pestis* is generally treatable with antimicrobials (Bonacorsi *et al.*, 1994), although acquired resistance has been observed in isolated cases in Madagascar (Galimand *et al.*, 2006); details of the plasmid that confers the multidrug-resistant phenotype have been published (Welch *et al.*, 2007).

A lot of work has focused on genomic and phenotypic differences between *Y. pestis* and *Y. pseudotuberculosis*. One of the most striking differences is the presence in most *Y. pestis* of two plasmids, pMT1 and pCP1, that are absent from all *Y. pseudotuberculosis* (Hu *et al.*, 1998). *Y. pseudotuberculosis* is still pathogenic (Rosqvist *et al.*, 1988), and *Y. pestis* and *Y. pseudotuberculosis* share genes associated with pathogenesis, including the pYV plasmid (also known as pCD1) and the *ail* locus (Reuter *et al.*, 2014), which is associated with attachment and invasion. A filamentous phage, YpfΦ, has been characterized in some *Y. pestis*, but is absent from all known *Y. pseudotuberculosis* genomes (Derbise & Carniel, 2014); this phage has been associated with dissemination in mice (Derbise *et al.*, 2007). Additionally, mutations have been described in *Y. pestis* compared with *Y. pseudotuberculosis* that have led to flea-borne transmission of *Y. pestis* (Sun *et al.*, 2014) due to enhanced biofilm formation.

In this study, we compared the entire pan-genomes of both species with the large-scale blast score ratio (LS-BSR) pipeline (Sahl *et al.*, 2014) to better understand the distribution and conservation of identified coding regions. Smaller-scale comparisons between *Y. pestis* and *Y. pseudotuberculosis* have been published (Achtman *et al.*, 1999; Chain *et al.*, 2004; Duan *et al.*, 2014; Pouillot *et al.*, 2008), but LS-BSR on a large set of genomes enabled us to rapidly compare the genomic content between species. These comparisons enabled us to identify fine-scale resolution in the global propagation of the *Y. pestis* clone. We also screened previously published genes against this comprehensive dataset to determine how well these previous studies captured the gene distribution across *Y. pestis* and *Y. pseudotuberculosis*.

## Methods

### Genomes analysed

Except where noted otherwise, our comparisons included 133 *Y. pestis* genomes and 13 *Y. pseudotuberculosis* genomes, as well as five plasmids (Table S1, available in the online Supplementary Material). To improve the assembly contiguity for 118 genomes recently published (Cui *et al.*, 2013) and assembled with Velvet (Zerbino & Birney, 2008), raw reads were downloaded and assembled with SPAdes version 3.1.0 (Bankevich *et al.*, 2012). This method improved the contiguity of all but one assembly and increased the assembly size (Table S2). The SPAdes assemblies are publicly available and named by the Sequence Read Archive accession number (https://github.com/jasonsahl/YP_genomes.git).

### Core genome single nucleotide polymorphism (SNP) phylogeny

Genome assemblies from external genomes were aligned against the finished genome of Colorado 92 (CO92) (GenBank accession number NC_003143) with NUCmer (Delcher *et al.*, 2003). The reference was also aligned against itself to identify duplicated regions, which were then removed for SNP comparisons. SNPs were identified from genome assemblies by a direct mapping of each query base to the corresponding reference base. Raw reads from a previously published study (Cui *et al.*, 2013) were mapped to CO92 with bwa-mem (Li, 2013) and SNPs were called with the UnifiedGenotyper method in the Genome Analysis Toolkit (DePristo *et al.*, 2011; McKenna *et al.*, 2010); any SNP with a coverage of < 10 or an allele proportion < 90 % were filtered from downstream analyses. These methods were wrapped by the Northern Arizona SNP Pipeline (NASP) (http://tgennorth.github.io/NASP/) (Engelthaler *et al.*, 2014). A phylogeny was inferred on the concatenated SNP alignment using RAxML version 8 (Stamatakis, 2014) and the ASC_GTRGAMMA model (Lewis correction) that incorporates an ascertainment bias correction. The retention index (RI) was calculated with phangorn (Schliep, 2011).

### Comparative genomics

All coding regions were predicted from all genome assemblies with Prodigal (Hyatt *et al.*, 2010). These coding regions were concatenated, then clustered and de-replicated using usearch (Edgar, 2010) at a pairwise identity of 0.9. Each cluster representative, or the cluster centroid, was then aligned against each genome using either tblastn (Altschul *et al.*, 1997) or blat (Kent, 2002) in conjunction with LS-BSR (Sahl *et al.*, 2014); the different methods were used to examine the conservation of either nucleotide or peptide sequences. LS-BSR values can range from 0 (no alignment) to 1 (100 % identity). To identify correlations between coding region conservation and metadata (e.g. isolation location), LS-BSR values were first multiplied by 100 in order to convert all float values to integers and these adjusted BSR (Rasko *et al.*, 2005) values were then correlated with categorical data using a Kruskal–Wallis test implemented in qiime version 1.9.0 (Caporaso *et al.*, 2010), which also incorporated false detection rate (FDR) adjusted *P* values. Categories tested included: branch location within the *Y. pestis* phylogeny (0–4), subclade in the *Y. pestis* phylogeny (e.g. 0.PE4Ba), country of strain origin, latitude of strain origin, number of nodes to the most recent common ancestor (MRCA; *Y. pseudotuberculosis*) and number of SNPs to the MRCA. Coding regions that showed positive correlations (FDR *P* < 0.05) were aligned against the GenBank (Benson *et al.*, 2012) nr database with tblastn (Altschul *et al.*, 1997) for annotation. Pan-genome stats were calculated with the pan_genome_stats.py script in the LS-BSR repository (https://github.com/jasonsahl/LS-BSR/tree/master/tools).

### Distribution of annotated genes

From reviewing the relevant literature, we identified a panel of genes associated with *Y. pestis* virulence (Chain *et al.*, 2004; Derbise *et al.*, 2007; Lillard *et al.*, 1997; Pouillot *et al.*, 2008; Radnedge *et al.*, 2002; Reuter *et al.*, 2014; Schubert *et al.*, 1998; Sodeinde *et al.*, 1992; Sun *et al.*, 2014) (Table S3), and screened these for conservation across 133 contemporary genomes of *Y. pestis* and 13 *Y. pseudotuberculosis* genomes using LS-BSR in conjunction with tblastn. In general, a gene was considered conserved if it had BSR>0.8, which is ∼80 % peptide identity over 100 % of the peptide length (Rasko *et al.*, 2008). The conservation of genes was visualized as a heatmap correlated with a phylogeny using the Interactive Tree Of Life (iTOL) (Letunic & Bork, 2007).

### Read mapping to confirm gene presence/absence

To confirm the presence or absence of specific coding regions, raw sequence reads were mapped to the reference sequence with bwa-mem. The coverage across each base was calculated with the GenomeCoverageBed method in BEDTools (Quinlan & Hall, 2010). Presence was dependent on the breadth of coverage across the reference at a minimum depth of coverage (DOC) (default value of × 3 was used); a script to wrap these methods is available (https://gist.github.com/3206314afe510b9e2cbb.git). Differences in mean DOC were visualized with Circos (Krzywinski *et al.*, 2009).

### Multiple sequence alignment analysis

For cases where individual mutations were investigated, the gene sequence was pulled out of all genome assemblies directly from blastn alignments (Altschul *et al.*, 1990), aligned with muscle (Edgar, 2004) and visualized with Jalview (Waterhouse *et al.*, 2009).

## Results

### Core genome phylogeny

**Fig. 1.**
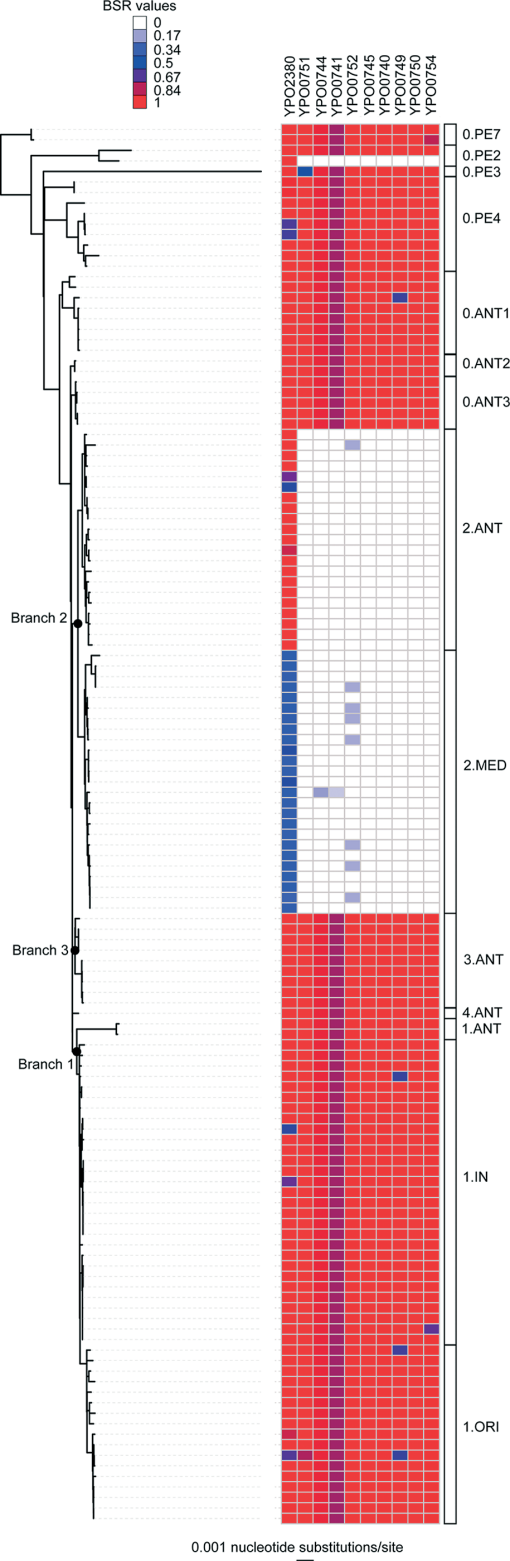
Loss of genes across Branch 2 genomes across the *Y. pestis* phylogeny. Differentially conserved genes were identified through associations of metadata and the LS-BSR (Sahl *et al.*, 2014) matrix using qiime (Caporaso *et al.*, 2010). The phylogeny was inferred with RAxML (Stamatakis, 2014) on a concatenation of SNPs identified with NASP (Engelthaler *et al.*, 2014). The phylogeny was associated with the LS-BSR values using iTOL (Letunic & Bork, 2007).

A core genome SNP phylogeny was inferred to demonstrate the relationship between *Y. pestis* genomes screened in the current study. The resulting phylogeny (Fig. 1) was similar in clade membership to a previous study that used the same set of genomes (Cui *et al.*, 2013). The RI (Farris, 1989) of the concatenated SNP alignment was 0.99, indicating very little homoplasy in the underlying data. A phylogeny was also inferred from an alignment including 13 *Y. pseudotuberculosis* genomes as well as the 133 *Y. pestis* genomes, rooted with *Yersinia enterolitica* 8081 (GenBank accession number NC_008800) (Fig. S1); for visualization, SNPs specific to *Y. enterolitica* were removed from the alignment. The phylogeny demonstrated the increased phylogenetic diversity in *Y. pseudotuberculosis* relative to *Y. pestis*. The RI of this alignment was 0.85, demonstrating significant homoplasy, likely resulting from recombination.

### *In silico* screen of the *Y. pseudotuberculosis* pan-genome across 133 *Y. pestis* genomes

**Fig. 2.**
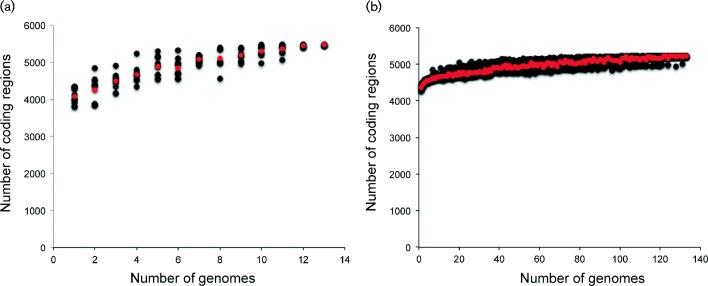
Pan-genome plots demonstrating the acquisition of genes with additional genomic sequencing in (a) *Y. pseudotuberculosis* and (b) *Y. pestis*. For each analysis, a number of randomly selected genomes were parsed at different genome depths from the LS-BSR (Sahl *et al.*, 2014) matrix, and the number of coding regions with BSR>0.8 in all genomes was identified and plotted.

To understand the pan-genome differences between *Y. pseudotuberculosis* and *Y. pestis*, the pan-genome was identified with LS-BSR for all *Y. pseudotuberculosis* genomes (*n* = 13), resulting in 5469 unique coding regions (Fig. 2a). Of these regions, seven were found to be highly conserved (BSR>0.8) in all *Y. pseudotuberculosis* genomes and missing (BSR < 0.4) from all *Y. pestis* genomes (Table 1); this demonstrated that most of the *Y. pseudotuberculosis* core genome was also conserved in at least one *Y. pestis* genome. *Y. pestis* has been associated with genome reduction (Chain *et al.*, 2006) based on host adaptation, but this small number of regions represents either the limited number of ancestral genes that are no longer required by *Y. pestis*, genes that are no longer needed but have not been purged or the relatively short time since the split between *Y. pestis* and *Y. pseudotuberculosis*.

**Table 1:** Annotation of coding regions unique to either *Y. pestis* or *Y. pseudotuberculosis*

Annotation	GenBank accession no.	Group
Hypothetical protein	WP_038824878	*Y. pseudotuberculosis*
Uracil transporter	WP_038824557	*Y. pseudotuberculosis*
Hypothetical protein	WP_011192493	*Y. pseudotuberculosis*
Aldehyde dehydrogenase	WP_011192485	*Y. pseudotuberculosis*
TonB-dependent vitamin B12 receptor	ACA70332	*Y. pseudotuberculosis*
Hypothetical protein	WP_038824929	*Y. pseudotuberculosis*
Hypothetical protein	ABS48865	*Y. pseudotuberculosis*
Hypothetical protein	YPO0396	*Y. pestis*
Integrase	YOP2083	*Y. pestis*
Hypothetical protein	YPO0387	*Y. pestis*
Hypothetical protein	YPO0388	*Y. pestis*
Hypothetical protein	YPO3437	*Y. pestis*
XRE family transcriptional regulator	WP_002214362	*Y. pestis*
Transcriptional regulator	YPO4031	*Y. pestis*
Transposase	WP_002214360	*Y. pestis*
Outer membrane receptor	YPO3910	*Y. pestis*
Hypothetical protein	YPO3948	*Y. pestis*
Hypothetical protein	YPO0394a	*Y. pestis*
Hypothetical protein	YPO0394	*Y. pestis*
Integrase	YPO4033	*Y. pestis*
Pseudogene	YPO4029	*Y. pestis*
Hypothetical protein	YPO0397	*Y. pestis*

The pan-genome of *Y. pseudotuberculosis* was screened across 133 *Y. pestis* genomes (Table S1) using the LS-BSR pipeline. Of all coding regions screened, 21 were found to have significantly lower BSR values (fdr adjusted *P* < 0.05) among Branch 2 genomes in the *Y. pestis* phylogeny when compared with other branches (Fig. 1), indicating loss of these coding regions since evolving from *Y. pseudotuberculosis*. Annotation of these regions using the CO92 annotation demonstrated that 19 out of 21 were known products, many involved with flagellar composition and function (Table 2); many of these coding regions have also been lost in Pestoides F (Fig. 1). One coding region was conserved in the 2.ANT lineage, but was absent from the 2.MED lineage (Fig. 1). Annotation of this sequence is associated with a toxin described in *Y. pseudotuberculosis* (locus tag WP_011192526); similar types of genes have recently been described as atypical toxins (Koskiniemi *et al.*, 2013). The 2.MED lineage contains genomes collected over a long time frame (1958–2006), with almost all of them isolated from China, suggesting local adaptation, niche specialization or a lack of sampling from other parts of the world.

**Table 2:** Annotation of regions lost by Branch 2 genomes

Locus	Annotation	Locus tag
*fliD*	Flagellar hook-associated protein	YPO0740
*fliS*	Flagellar protein	YPO0741
YPO0742	Hypothetical protein	YPO0742
YPO0744	Flagellar biogenesis protein	YPO0744
*fliA*	Flagellar biosynthesis sigma factor	YPO0745
*motA*	Flagellar motor protein MotA	YPO0746
*motB*	Hypothetical protein	YPO0747
YPO0749	Hypothetical protein	YPO0749
YPO0750	Hypothetical protein	YPO0750
YPO0751	Hypothetical protein	YPO0751
YPO0754	Hypothetical protein	YPO0754
YPO1380	MFS family transporter protein	YPO1380
YPO2315	Hypothetical protein	YPO2315
*Pcp*	Lipoprotein	YPO2373
YPO2375	Aldo/keto reductase	YPO2375
YPO2376	Hypothetical protein	YPO2376
*sepC*	Insecticial toxin	YPO2380
YPO2493	Dioxygenase subunit alpha	YPO2493

### *In silico* screen of the *Y. pestis* pan-genome across *Y. pseudotuberculosis* genomes

Based on default values in LS-BSR, the *Y. pestis* pan-genome was found to consist of 5227 unique coding regions (Fig. 2b). A screen of these coding regions against all *Y. pseudotuberculosis* genomes demonstrated that there were 15 coding regions present in all *Y. pestis* genomes (BSR>0.8) and missing from all *Y. pseudotuberculosis* genomes (BSR < 0.4) (Table 1); none of these regions was associated with any of the known *Y. pestis* plasmids. In the pan-genome, there were 725 coding regions that were conserved in one or more *Y. pestis* genome and missing from all *Y. pseudotuberculosis* genomes. Of the total *Y. pestis*-specific coding regions, 156 of these coding regions were associated with *Y. pestis* plasmids (BSR>0.8). Of the remaining coding regions, 294 were only conserved in a single genome, suggesting possible contamination, sequencing artefacts or horizontally acquired genomic regions. For example, 228 of these unique regions were identified in the draft genome assembly of Orientalis IP275 and are most likely associated with a plasmid that confers resistance to multiple antimicrobials (Welch *et al.*, 2007). The remaining *Y. pestis*-specific coding regions (*n* = 275) were conserved in two or more genomes (Table S4).

Chain *et al.* (2004) identified a total of 112 *Y. pestis*-specific genes and screened them across a panel of 19 *Y. pestis* strains. They found 32 of these regions to be conserved in all of their *Y. pestis* strains, but in none of the nine screened *Y. pseudotuberculosis* strains. An *in silico* screen of these regions (Table S3) against our collection of genomes demonstrated that only five genes were completely conserved across the *Y. pestis* phylogeny (BSR>0.8 in 133 genomes) (Table S5). One of the regions (WP_002213869) was conserved (BSR = 1) in two *Y. pseudotuberculosis* genomes (B-7194, CBKS0000000; B-7195, CBKR00000000) in our collection.

### *In silico* screen of previously annotated genes

**Fig. 3.**
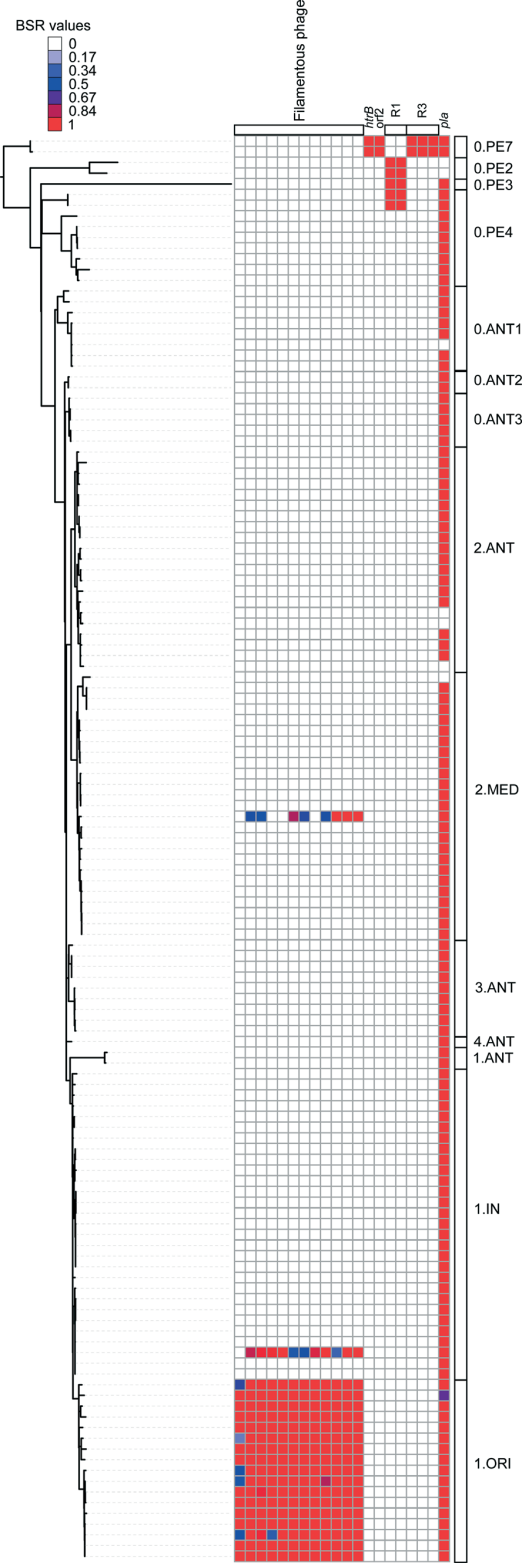
Variable distribution of previously characterized genes across the *Y. pestis* phylogeny (Table S3). The phylogeny was inferred with RAxML (Stamatakis, 2014) on a concatenation of SNPs identified with NASP (Engelthaler *et al.*, 2014). The phylogeny was associated with the LS-BSR values using iTOL (Letunic & Bork, 2007).

LS-BSR analysis demonstrated that the 11 genes from a previously described *Y. pestis* filamentous phage (Derbise *et al.*, 2007) associated with mouse dissemination were conserved in all genomes within the 1.ORI group (Fig. 3); this region was also identified in one genome (YN472) from the 1.IN3 group and a portion of the phage was identified in a genome (H1958004) from the 2.MED group (Fig. 3). A read mapping analysis confirmed the presence of portions of this phage in these two genomes, although the DOC was significantly lower compared with the coverage in 1.ORI genomes. For example, in genome fig1946001 from the 1.ORI group, the mean DOC across the genome was × 58 and the coverage across the phage was × 243. In contrast, the DOC across the genome of YN472 from the 1.IN group was × 108 and only × 5 across the phage. This lower level of coverage could be due to copy number variation or cross-sample contamination during sequencing and would need to be confirmed with targeted assays. If confirmed, the presence of this phage in the 1.IN3 group would suggest that this lineage gave rise to 1.ORI, as has been proposed previously (Cui *et al.*, 2013). All coding regions from the filamentous phage were missing (BSR < 0.4) from all *Y. pseudotuberculosis* genomes. Li *et al.* (2008) identified three of these phage ORFs in a strain that was included in our panel (A1956001), but our analysis identified these ORFs as missing (BSR = 0.0) in the genome assembly. We validated the absence of these ORFs in this assembly with read mapping.

From a pathogenesis perspective, one of the genomic regions associated with virulence is the HPI (high pathogenicity island), a component of the *pgm* locus (Schubert *et al.*, 1998). Prodigal was used to predict coding regions from this region (AL031866) and all coding regions (*n* = 93) were screened against all genomes with LS-BSR. The results demonstrate that a large section (∼65 coding regions) of the *pgm* locus was consistently missing from multiple (∼30) *Y. pestis* genomes (Fig. S2); the *pgm* was largely conserved across screened *Y. pseudotuberculosis* genomes. A subset of the *pgm* locus, the *hmsHFRS* operon, was reported to be necessary for the synthesis of *Y. pestis* biofilm in fleas (Sun *et al.*, 2014) and therefore flea-borne transmission of the pathogen. Sun *et al.* (2014) further stated that both *Y. pestis* and *Y. pseudotuberculosis* contained a fully functional *hmsHFRS* operon. However, our LS-BSR analysis of the four *hmsHFRS* loci (*hmsH*, *hmsF*, *hmsR* and *hmsS*; GenBank accession number U22837) (Lillard *et al.*, 1997) demonstrated that these genes were conserved in only a portion (106/133) of our *Y. pestis* panel. In contrast, these four loci were present in all of our screened *Y. pseudotuberculosis* genomes.

Reuter *et al.* (2014) reported that the chromosomal type III secretion system (abbreviated Ygt for the *Yersinia* genus type III secretion system) is present in all *Yersinia* species, but in the process of being lost from the pathogenic lineages (Reuter *et al.*, 2014). They screened 32 regions (Table S3) of Ygt across three and 31 *Y. pestis* and *Y. pseudotuberculosis* strains, respectively, and found them to be variably distributed across these two species (range 0–84.68 %; mean ± sd 45.68 ± 32.45 %). In our global LS-BSR analysis of these 32 regions in our panel of genomes, we found 30 of 32 regions to be missing (BSR < 0.1) from all screened *Y. pestis* and *Y. pseudotuberculosis* genomes. The remaining two regions (strain YE21202, coordinates 3177984:3178778 and 3178768:3179844) were not highly conserved across the majority of genomes in both species (BSR < 0.5). These two regions were categorized as putative type III secretion proteins.

The *pla* gene (locus tag YPPCP1.07) on the pPCP1 plasmid encodes a cell surface protease/plasminogen activator that is essential for invasiveness at the flea-bite site following *Y. pestis* transmission (Sodeinde *et al.*, 1992; Sun *et al.*, 2014). The LS-BSR analysis demonstrated the conservation (BSR = 1) of this gene in most contemporary *Y. pestis* strains (*n* = 125/133) (Fig. 3); the absence of this locus in some genomes may represent the loss of the pPCP1 plasmid due to laboratory passage (Perry & Fetherston, 1997). The *pla* gene was missing from all *Y. pseudotuberculosis* genomes surveyed (BSR = 0).

Pouillot *et al.* (2008) screened 47 *Y. pestis* and 31 *Y. pseudotuberculosis* strains for five regions and four ORFs (Table S3) that were specific to *Y. pseudotuberculosis*. They concluded that despite their loss in *Y. pestis*, five out of nine of these (ORF2, ORF3, ORF4, R1, R3) were important to survival, growth or virulence in *Y. pseudotuberculosis*. An *in silico* screen of these five loci demonstrated that R1 was conserved in *Y. pestis* Branches 0.PE2, 0.PE3 and 0.PE4A only, suggesting independent loss in both 0.PE7 and all other branches of *Y. pestis* (Fig. 3). ORF2 was present in Branch 0.PE7 only (Fig. 3), suggesting that the deletion of this region happened after the split between *Y. pseudotuberculosis* and *Y. pestis*. In *Y. pseudotuberculosis*, ORF2 and R1 were highly conserved (BSR>0.98) across all sequenced genomes, but ORF3 and ORF4 were only conserved (BSR>0.8) in a portion of *Y. pseudotuberculosis* genomes (ORF3 in four of 13; ORF4 in seven of 13).

Chain *et al.* (2004) reported the loss of a lipid A acyltransferase gene *htrB* (locus tag YPTB2490) in all *Y. pestis* since its divergence from *Y. pseudotuberculosis.* However, our LS-BSR analysis demonstrated that this gene was conserved in the two most ancestral contemporary strains of *Y. pestis* on Branch 0.PE7 (620024 and CMCC05009) (Fig. 3). This gene was also highly conserved in all *Y. pseudotuberculosis* genomes (BSR ≥ 0.99). Chain *et al.* (2004) also reported nine coding regions (YPTB3450–YPTB3459) conserved in *Y. pseudotuberculosis*, representing a region that encodes several haemolysin genes absent from *Y. pestis*. Our *in silico* screen of these nine regions in our panel of *Y. pestis* assemblies demonstrated variation in the conservation of these regions across assemblies in both species (Table S6). Five of these regions were missing across all screened *Y. pestis* genomes (BSR = 0) and the remaining four demonstrate low homology across *Y. pestis* genomes (BSR = 0–0.86). In our screened *Y. pseudotuberculosis* genomes, all of these regions were completely conserved (BSR = 1) in only two of 13 genomes.

Chain *et al.* (2004) also reported frameshift mutations in two virulence factor genes characterized in *Y. pestis*, *srfA* and *srfB* (YPTB2212 and YPTB2213, respectively, both discontinued in the National Center for Biotechnology Information database) that are conserved in *Y. pseudotuberculosis*. The authors speculated that if these mutations influence protein function, these loci might be involved in species-specific virulence. Our *in silico* screen of these regions demonstrated that both loci are conserved in all *Y. pestis* genomes, although small deletions observed in the multiple sequence alignment could result in frameshift mutations; these deletions were observed in only a portion of *Y. pseudotuberculosis* genomes (Fig. S3). In *Y. pseudotuberculosis*, *srfA* was conserved in all screened genomes (mean BSR = 0.98), but *srfB* was not conserved (BSR < 0.51) in six out of 13 genomes.

In their 2014 paper, Sun *et al.* (2014) detailed three loss-of-function mutations that increased the transmissibility of *Y. pestis* via flea bite. We screened both the *Y. pestis* Kim10 and *Y. pseudotuberculosis* IP32953 homologues for PDE2, PDE3 and *rcsA* (Table S3). Whilst Sun *et al.* (2014) reported that PDE3 was conserved in all *Y. pestis* genomes except for Angola and Pestoides F strains, we found that PDE3 was also missing from genome Antigua UG05-0454, Branch 0.PE2 and Branch 0.ANT3 (Fig. S4); we also found that PDE3 was missing (BSR < 0.2) from six of 13 *Y. pseudotuberculosis* genomes. Sun *et al.* (2014) also reported a 30 bp tandem duplication in *rcsA* that was present in all *Y. pestis* except for Branch 0 strain Pestoides A. We screened the *Y. pestis* version of this gene (YP02449) in a multiple sequence alignment and found the duplication to be missing in additional genomes across the *Y. pestis* phylogeny (C1975003, M0000002, SHAN11, YN1683, Antigua, Antigua UG05, CMCC96007, CMCC11001, CMCC03001) (Fig. S5); the tandem duplication was confirmed to be missing from all *Y. pseudotuberculosis* genomes. Whilst PDE2 was highly conserved across all *Y. pestis* genomes, it was missing from one *Y. pseudotuberculosis* genome (B-6863), although this may be have been due to an assembly error. The reported frameshift was found in all *Y. pestis* genomes and none of the *Y. pseudotuberculosis* genomes.

Difference region (DFR) 4 is a 15 kb genomic island that is known to be lost in some *Y. pestis* strains (i.e. CO92) and contains genes that may play a role in virulence. Radnedge *et al.* (2002) investigated the presence of DFR4 in 78 *Y. pestis* and four *Y. pseudotuberculosis* strains, and found it to be conserved in only 13 of the *Y. pestis* strains but in all four *Y. pseudotuberculosis* strains. LS-BSR of 16 coding regions of DFR4 demonstrated them all to be missing from 48 genomes, including the entire 0.PE2, 0.PE3, 0.PE4A, 1.IN3, 1.ORI and 2.MED1 branches. One coding region (AAL27378) was not highly conserved in all 133 screened *Y. pestis* genomes (BSR < 0.7) and one (AAL27386) was conserved in 27 (33 %) of the genomes; the remaining 14 coding regions were found in 85 (64 %) *Y. pestis* genomes. Fourteen out of 16 coding regions were conserved in all screened *Y. pseudotuberculosis* genomes, whilst the other two coding regions were variably distributed.

Our LS-BSR analysis of the remaining DFRs (DFR1–3, 5 and 6) described by Radnedge *et al.* (2002) demonstrated that DFR5 was completely lost in all *Y. pestis* genomes (Table 3). In DFR2, three out of four coding regions had been lost across all *Y. pestis* genomes. For the remaining DFRs, two out of three regions of each DFR were demonstrated to be lost across all *Y. pestis* genomes, but the third was found to be conserved in a portion of *Y. pestis* genomes: 65 % in DFR1 (AF333796), 87 % in DFR3 (AF333802), 80 % in DFR6 (AF333812) and 99 % in DFR2 (AF333801). In *Y. pseudotuberculosis*, DFR5 was missing from all screened genomes and aff333802 of DFR3 was conserved in all *Y. pseudotuberculosis* genomes. However, similar to what was found in *Y. pestis*, three out of four regions in DFR2 were missing across all *Y. pseudotuberculosis* genomes and two out of three regions of the remaining DFRs were missing across all *Y. pseudotuberculosis* genomes. The remaining one region within each DFR was present in 46 % in DFR1 (AF333796), 54 % in DFR2 (AF333801) and 92 % in DFR6 (AF333812) across all *Y. pseudotuberculosis* genomes.

**Table 3:** Comparison of screening results between previous studies and the current study

Gene/region	Locus tag(s)	Reference	Previous results (%)		Results from current study (%)	
			*Y. pestis*	*Y. pseudotuberculosis*	Y. pestis^*^ (*n* = 133)	Y. pseudotuberculosis^*^ (*n* = 13)
Filamentous phage	YPO2271–YPO2280	Derbise *et al.* (2007)	na	na	100 (1.ORI)	0
*Y. pestis* specific genes	See Table S3	Chain *et al.* (2004)	100 (*n* = 19)	0 (*n* = 9)	18–100	2–15
Ygt	See Table S3	Reuter *et al.* (2014)	na	na	0	0
*hmsHFRS*	U22837	Sun *et al.* (2014)	100	100	80	100
*Pla*	YPPCP1.07	na	na	na	94	0
*Y. pseudotuberculosisSpecific genes*	See Table S3	Pouillot *et al.* (2008)	0 (*n* = 47)	100 (*n* = 31)	0–14	0–100
*htrB*	YPTB2490	Chain *et al.* (2004)	0 (*n* = 19)	100 (*n* = 9)	1.50	100
Haemolysin	YPTB3450–YPTB3459	Chain *et al.* (2004)	0 (*n* = 19)	100 (*n* = 9)	0–9	23–46
DFR4	AF426171	Radnedge *et al.* (2002)	17 (*n* = 78)	100 (*n* = 4)	0–85	38–100
DFR1	See Table S3	Radnedge *et al.* (2002)	79 (*n* = 78)	100 (*n* = 4)	22	14
DFR2	AF333798–AF333801	Radnedge *et al.* (2002)	97 (*n* = 78)	0 (*n* = 4)	25	16
DFR3	AF333802–AF333804	Radnedge *et al.* (2002)	72 (*n* = 78)	100 (*n* = 4)	28	32
DFR5	AF333808–AF333810	Radnedge *et al.* (2002)	68 (*n* = 78)	0 (*n* = 4)	0	0
DFR6	AF333811–AF333813	Radnedge *et al.* (2002)	90 (*n* = 78)	100 (*n* = 4)	26	30
PDE2 frameshift	YPTB1308	Sun *et al.* (2014)	100 (*n* = 29)	0	100	0
PDE3 presence	YPTB3308	Sun *et al.* (2014)	93 (*n* = 29)	na	93	54
*rcsA* duplication	YPTB2486	Sun *et al.* (2014)	97 (*n* = 29)	0	93	0
*ail* attachment locus	YE1820	Reuter *et al.* (2014)	70–90 (*n* = 3)	70–90 (*n* = 31)	0	0
*Ymt*	Y1069	Sun *et al.* (2014)	na	na	0–100	0
*srf*A frameshift	YPTB2212	Chain *et al.* (2004)	na	na	100	100
*srfB* frameshift	YPTB2213	Chain *et al.* (2004)	na	na	100	54

na, Results not reported.

* Presence based on BSR>0.8.

Although the focus of this work was on contemporary strains, ancient genomes are now available from the first and second pandemics (Table S1); the genome sequences from the second pandemic were generated with a capture array using CO92 as the reference, which may generate false negatives for genes not present in this genome. Reads from ancient genomes were mapped across the genes screened in this study and variable distribution was observed (Table S7). With ancient genomes, the low and variable coverage prohibits any firm conclusions about the presence or absence of screened genes.

### Distribution of plasmids across *Y. pestis*

**Fig. 4.**
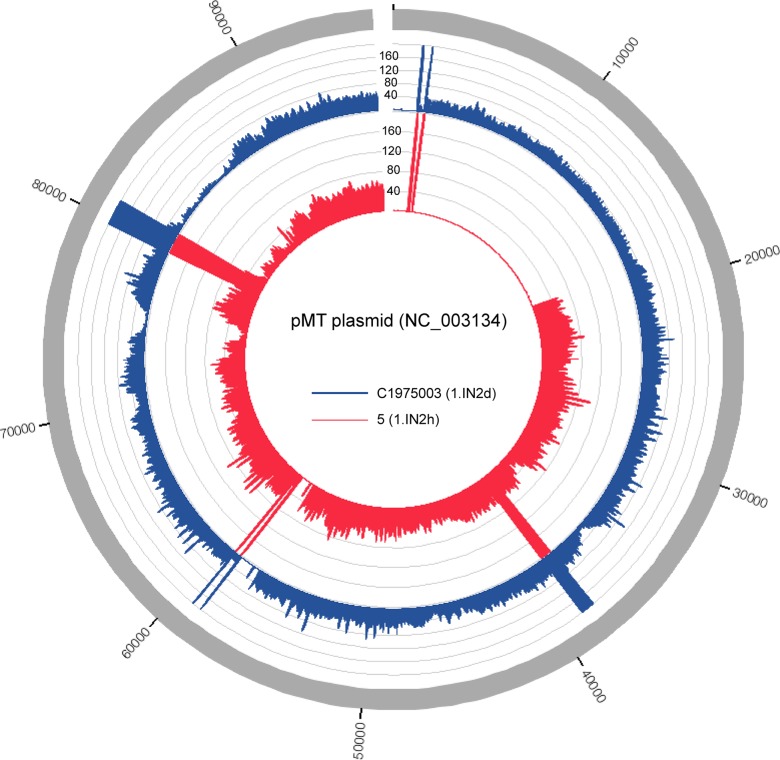
Variable distribution of raw sequence reads from two 1.IN genomes across the pMT plasmid. Reads were mapped to the plasmid with bwa-mem (Li, 2013) and the mean DOC was calculated with the GenomeCoverageBed method in BEDTools (Quinlan & Hall, 2010). The differences in DOC were visualized with Circos (Krzywinski *et al.*, 2009).

One of the defining features of *Y. pestis* is the acquisition of multiple plasmids that are absent from *Y. pseudotuberculosis*. We screened each coding region for each plasmid (Table S1) against 133 *Y. pestis* genomes to look at their distribution; by only considering coding regions, we could make no comment about the synteny of plasmid structure between different isolates. The results demonstrated that pPCP1 and pCD1 are broadly conserved across *Y. pestis* genomes (Table S8), although they have been intermittently lost in select genomes, likely due to laboratory passage. The pMT plasmid was also intermittently conserved, although a large region (∼16.7 kb) of the plasmid appears to be missing (Supplementary Data File 1) in a monophyletic clade within the 1.IN2 group (1.IN2h–1IN2p); annotation of missing coding regions was associated with phage-related genes. Deleted regions can easily be visualized by mapping reads across the plasmid between a genome with the region (*Y. pestis* C1975003) and a closely related genome missing the region (*Y. pestis* 5) (Fig. 4).

## Discussion

*Y. pestis* is the causative agent of plague and a recently derived clone of *Y. pseudotuberculosis*. The evolution of *Y. pestis* has been associated with the acquisition of virulence plasmids, mutations that allow for transmission via flea bites and the loss of genes due to host adaptation. In this study, we used publicly available genomes to comprehensively characterize the gain, loss and variable composition in the *Y. pestis* pan-genome following its separation from the common ancestor with *Y. pseudotuberculosis*.

The evolution of *Y. pestis* has been described as largely clonal (Achtman, 2008), likely due to the specialized habitat of *Y. pestis*. The core genome SNP phylogeny of *Y. pestis* confirmed this observation, with very little homoplasy detected. However, an analysis of the pan-genome demonstrated that the gene content can be widely variable and does not necessarily resemble the evolution of other clonal pathogens, such as *Bacillus anthracis* (Van Ert *et al.*, 2007). Therefore, the evolution of *Y. pestis* can be thought of as a clonal expansion with regard to the core genome, with a pan-genome characterized by differential gene loss and acquisition of mobile genetic elements including plasmids and bacteriophages.

A comparison of the pan-genomes between *Y. pestis* and *Y. pseudotuberculosis* demonstrated that the *Y. pseudotuberculosis* pan-genome is larger (Fig. 2), suggesting either that coding regions have been lost in *Y. pestis* due to adaptation or the genomic diversity of *Y. pseudotuberculosis* is much greater. Comparative genomics identified coding regions both lost by all *Y. pestis* compared with *Y. pseudotuberculosis*, as well as coding regions unique to *Y. pestis*. Whilst some of these regions were associated with acquired plasmids, others were associated with the chromosome, suggesting acquisition after the differentiation from *Y. pseudotuberculosis*. These unique regions could serve as diagnostic markers for either field or clinical differentiation between the two species. These species-specific markers did not overlap well with previously published markers, suggesting that our larger sample set provides greater resolution into the dynamics of pan-genome structures. Annotation of *Y. pestis* specific coding regions was largely associated with hypothetical proteins (Table 2), which presents targets for functional studies investigating the pathogenesis, evolution and spread of *Y. pestis*.

Other studies have investigated the genomic differences between *Y. pestis* and *Y. pseudotuberculosis* based on smaller-scale analyses (Achtman *et al.*, 1999; Chain *et al.*, 2004; Duan *et al.*, 2014; Pouillot *et al.*, 2008). Our results demonstrate that several genomic features reported to be missing from *Y. pestis*, such as *htrB*, are conserved in a subset of *Y. pestis* genomes (Fig. 3). Other genomic features, such as coding regions of the pMT plasmid, are variably conserved (Fig. 4), but this result was concordant with the core genome SNP phylogeny. These observations would not have been possible without analysing a comprehensive panel of *Y. pestis* genomes. Additional sequencing of *Y. pestis* isolates from under-sampled geographical regions may further refine gene conservation and distribution in *Y. pestis*.

In addition to screening previously characterized genes, we also performed a *de novo* analysis to identify genes that were associated with different categorical data. The most associated pattern of gene conservation was in Branch 2 genomes, where several genes have been lost compared to other contemporary *Y. pestis* isolates. Many of these genes were associated with the flagellum, which suggests that these genes are being lost because *Y. pestis* does not appear to be motile (Brubaker, 1991). Continued surveillance of this pathogen will determine if these genes will be lost by additional lineages in the future.

A recent study characterized three mutations in *Y. pestis* compared with *Y. pseudotuberculosis* that result in enhanced biofilm formation and transmissibility (Sun *et al.*, 2014). However, our analysis demonstrated variation in the presence of these mutations in the *Y. pestis* panel screened, suggesting that the lack of these mutations does not completely shut down the transmissibility of these isolates, other mechanisms are also responsible for biofilm formation or these strains are still transmitted by fleas, but just less efficiently. These questions will need to be addressed with additional experimentation.

Overall, this study highlights the necessity of sequencing and analysing a large number of genomes from a given species to understand the distribution of genes, especially in relation to close near-neighbours of a given species. These types of comparative studies can focus functional studies that aim at understanding the global evolution of a given species. Although this study is focused on *Y. pestis*, the methods can be applied to any organism to characterize the gene flow, including gain and loss, across a comprehensive set of sequenced genomes.

## Supplementary Data

3098 Supplementary Data
