# Supplementary material for: Redefining the differences in gene content between Yersinia pestis and Yersinia pseudotuberculosis using large-scale comparative genomics
Source: Microb Genom. 2015 Aug 3;1(2):e000028. doi: 10.1099/mgen.0.000028 (PMC5320571; doi:10.1099/mgen.0.000028)

Table S1. Accession information for genomes screened in the current study

**Species**	**Strain ID**	**NCBI Genome Accession**	**Publication**
Y. pestis	12	ADOV00000000	Cui et al. 2012
Y. pestis	164	ADOW00000000	Cui et al. 2012
Y. pestis	2	ADOX00000000	Cui et al. 2012
Y. pestis	2330	ADOY00000000	Cui et al. 2012
Y. pestis	2504	ADOZ00000000	Cui et al. 2012
Y. pestis	2506	ADPA00000000	Cui et al. 2012
Y. pestis	2654	ADPB00000000	Cui et al. 2012
Y. pestis	30017	ADPC00000000	Cui et al. 2012
Y. pestis	34008	ADPD00000000	Cui et al. 2012
Y. pestis	34202	ADPE00000000	Cui et al. 2012
Y. pestis	351001	ADPF00000000	Cui et al. 2012
Y. pestis	42013	ADPG00000000	Cui et al. 2012
Y. pestis	42082	ADPH00000000	Cui et al. 2012
Y. pestis	42091	ADPI00000000	Cui et al. 2012
Y. pestis	42095	ADPJ00000000	Cui et al. 2012
Y. pestis	5	ADPK00000000	Cui et al. 2012
Y. pestis	5761	ADPL00000000	Cui et al. 2012
Y. pestis	620024	ADPM00000000	Cui et al. 2012
Y. pestis	7	ADPN00000000	Cui et al. 2012
Y. pestis	71021	ADPO00000000	Cui et al. 2012
Y. pestis	710317	ADPP00000000	Cui et al. 2012
Y. pestis	7338	ADPQ00000000	Cui et al. 2012
Y. pestis	735	ADPR00000000	Cui et al. 2012
Y. pestis	780441	ADPS00000000	Cui et al. 2012
Y. pestis	9	ADPT00000000	Cui et al. 2012
Y. pestis	91	ADPU00000000	Cui et al. 2012
Y. pestis	945	ADPV00000000	Cui et al. 2012
Y. pestis	970754	ADPW00000000	Cui et al. 2012
Y. pestis	A1956001	ADPX00000000	Cui et al. 2012
Y. pestis	A1973001	ADPY00000000	Cui et al. 2012
Y. pestis	C1975003	ADPZ00000000	Cui et al. 2012
Y. pestis	C1976001	ADQA00000000	Cui et al. 2012
Y. pestis	C1989001	ADQB00000000	Cui et al. 2012
Y. pestis	CMCC02041	ADQC00000000	Cui et al. 2012
Y. pestis	CMCC03001	ADQD00000000	Cui et al. 2012
Y. pestis	CMCC05009	ADQE00000000	Cui et al. 2012
Y. pestis	CMCC05013	ADQF00000000	Cui et al. 2012
Y. pestis	CMCC10012	ADQG00000000	Cui et al. 2012
Y. pestis	CMCC104003	ADQH00000000	Cui et al. 2012
Y. pestis	CMCC106002	ADQI00000000	Cui et al. 2012
Y. pestis	CMCC107004	ADQJ00000000	Cui et al. 2012
Y. pestis	CMCC11001	ADQK00000000	Cui et al. 2012
Y. pestis	CMCC114001	ADQL00000000	Cui et al. 2012
Y. pestis	CMCC12003	ADQM00000000	Cui et al. 2012
Y. pestis	CMCC125002	ADQN00000000	Cui et al. 2012
Y. pestis	CMCC18019	ADQO00000000	Cui et al. 2012
Y. pestis	CMCC21106	ADQP00000000	Cui et al. 2012
Y. pestis	CMCC27002	ADQQ00000000	Cui et al. 2012
Y. pestis	CMCC31004	ADQR00000000	Cui et al. 2012
Y. pestis	CMCC347001	ADQS00000000	Cui et al. 2012
Y. pestis	CMCC348002	ADQT00000000	Cui et al. 2012
Y. pestis	CMCC38001	ADQU00000000	Cui et al. 2012
Y. pestis	CMCC42007	ADQV00000000	Cui et al. 2012
Y. pestis	CMCC43032	ADQW00000000	Cui et al. 2012
Y. pestis	CMCC49003	ADQX00000000	Cui et al. 2012
Y. pestis	CMCC51020	ADQY00000000	Cui et al. 2012
Y. pestis	CMCC64001	ADQZ00000000	Cui et al. 2012
Y. pestis	CMCC640047	ADRA00000000	Cui et al. 2012
Y. pestis	CMCC67001	ADRB00000000	Cui et al. 2012
Y. pestis	CMCC71001	ADRC00000000	Cui et al. 2012
Y. pestis	CMCC8211	ADRD00000000	Cui et al. 2012
Y. pestis	CMCC84033	ADRE00000000	Cui et al. 2012
Y. pestis	CMCC84038	ADRF00000000	Cui et al. 2012
Y. pestis	CMCC84046	ADRG00000000	Cui et al. 2012
Y. pestis	CMCC87001	ADRH00000000	Cui et al. 2012
Y. pestis	CMCC90027	ADRI00000000	Cui et al. 2012
Y. pestis	CMCC91090	ADRJ00000000	Cui et al. 2012
Y. pestis	CMCC92004	ADRK00000000	Cui et al. 2012
Y. pestis	CMCC92010	ADRL00000000	Cui et al. 2012
Y. pestis	CMCC93014	ADRM00000000	Cui et al. 2012
Y. pestis	CMCC95001	ADRN00000000	Cui et al. 2012
Y. pestis	CMCC96001	ADRO00000000	Cui et al. 2012
Y. pestis	CMCC96007	ADRP00000000	Cui et al. 2012
Y. pestis	CMCC99103	ADRQ00000000	Cui et al. 2012
Y. pestis	CMCCK100001	ADRR00000000	Cui et al. 2012
Y. pestis	CMCCK110001	ADRS00000000	Cui et al. 2012
Y. pestis	CMCCN010025	ADRT00000000	Cui et al. 2012
Y. pestis	D1964001	ADRU00000000	Cui et al. 2012
Y. pestis	D1964002	ADRV00000000	Cui et al. 2012
Y. pestis	D1982001	ADRW00000000	Cui et al. 2012
Y. pestis	D1991004	ADRX00000000	Cui et al. 2012
Y. pestis	E1977001	ADRY00000000	Cui et al. 2012
Y. pestis	EV76	ADSA00000000	Cui et al. 2012
Y. pestis	F1946001	ADSB00000000	Cui et al. 2012
Y. pestis	F1954001	ADSC00000000	Cui et al. 2012
Y. pestis	F1984001	ADSD00000000	Cui et al. 2012
Y. pestis	G1996006	ADSE00000000	Cui et al. 2012
Y. pestis	G1996010	ADSF00000000	Cui et al. 2012
Y. pestis	G8786	ADSG00000000	Cui et al. 2012
Y. pestis	H1958004	ADSH00000000	Cui et al. 2012
Y. pestis	H1959004	ADSI00000000	Cui et al. 2012
Y. pestis	I160001	ADSJ00000000	Cui et al. 2012
Y. pestis	I1969003	ADSK00000000	Cui et al. 2012
Y. pestis	I1970005	ADSL00000000	Cui et al. 2012
Y. pestis	I1991001	ADSM00000000	Cui et al. 2012
Y. pestis	I1994006	ADSN00000000	Cui et al. 2012
Y. pestis	I2001001	ADSO00000000	Cui et al. 2012
Y. pestis	J1963002	ADSP00000000	Cui et al. 2012
Y. pestis	J1978002	ADSQ00000000	Cui et al. 2012
Y. pestis	K21985002	ADSS00000000	Cui et al. 2012
Y. pestis	M0000002	ADST00000000	Cui et al. 2012
Y. pestis	MGJZ11	ADSU00000000	Cui et al. 2012
Y. pestis	MGJZ12	ADSV00000000	Cui et al. 2012
Y. pestis	MGJZ3	ADSW00000000	Cui et al. 2012
Y. pestis	MGJZ6	ADSX00000000	Cui et al. 2012
Y. pestis	MGJZ7	ADSY00000000	Cui et al. 2012
Y. pestis	MGJZ9	ADSZ00000000	Cui et al. 2012
Y. pestis	SHAN11	ADTA00000000	Cui et al. 2012
Y. pestis	SHAN12	ADTB00000000	Cui et al. 2012
Y. pestis	YN1065	ADTC00000000	Cui et al. 2012
Y. pestis	YN1683	ADTD00000000	Cui et al. 2012
Y. pestis	YN2179	ADTE00000000	Cui et al. 2012
Y. pestis	YN2551	ADTF00000000	Cui et al. 2012
Y. pestis	YN2588	ADTG00000000	Cui et al. 2012
Y. pestis	YN472	ADTH00000000	Cui et al. 2012
Y. pestis	YN663	ADTI00000000	Cui et al. 2012
Y. pestis	K11973002	NZ_AAYT00000000	Cui et al. 2012
Y. pestis	E1979001	NZ_AAYV00000000	Cui et al. 2012
Y. pestis	CO92	AL590842	Parkhill et al. 2001
Y. pestis	KIM10+	NC_004088	Deng et al. 2002
Y. pestis	91001	NC_005810	Zhou et al. 2004
Y. pestis	Antiqua	NC_008150	Chain et al. 2006
Y. pestis	PestoidesF	NC_009381	Copeland et al. unpub
Y. pestis	Angola	NC_010159	Eppinger et al. 2010
Y. pestis	IP275	NZ_AAOS00000000	Morelli et al. 2010
Y. pestis	UG05	NZ_AAYR00000000	Morelli et al. 2010
Y. pestis	MG05	NZ_AAYS00000000	Morelli et al. 2010
Y. pestis	B42003004	NZ_AAYU00000000	Eppinger et al. 2009
Y. pestis	F1991016	NZ_ABAT00000000	Eppinger et al. 2009
Y. pestis	CA88	NZ_ABCD00000000	Plunkett et al. unpub
Y. pestis	Nepal516	NZ_ACNQ00000000	Chain et al. 2006
Y. pestis	India195	NZ_ACNR00000000	Plunkett et al. unpub
Y. pestis	PestoidesA	NZ_ACNT00000000	Plunkett et al. unpub
Y. pseudotuberculosis	B-6796	CAQT00000000	Platonov et al. unpub
Y. pseudotuberculosis	B-6862	CAQU00000000	Platonov et al. 2013
Y. pseudotuberculosis	B-6863	CAQV00000000	Platonov et al. 2013
Y. pseudotuberculosis	B-6864	CAQW00000000	Platonov et al. 2013
Y. pseudotuberculosis	B-6865	CAQX00000000	Platonov et al. 2013
Y. pseudotuberculosis	B-6866	CAQY00000000	Platonov et al. 2013
Y. pseudotuberculosis	B-7194	CBKS00000000	Blouin et al. 2013
Y. pseudotuberculosis	B-7195	CBKR00000000	Blouin et al. 2013
Y. pseudotuberculosis	IP31758	CP000720,CP000719,CP000718	Eppinger et al. 2007
Y. pseudotuberculosis	IP32953	BX936398,BX936400,BX936399	Chain et al. 2004
Y. pseudotuberculosis	NBRC105692	BAUR00000000	Yoshida et al. unpub
Y. pseudotuberculosis	PB1/+	CP001048,CP001049	Challacombe et al. unpub
Y. pseudotuberculosis	YPIII	CP000950	Challacombe et al. unpub
Y. pestis plasmid pCD1 plasmid	CO92	AL117189	Parkhill et al. 1999
Y. pestis plasmid pMT1 plasmid	CO92	AL117211	Prentice et al. 2001
Y. pestis plasmid pCP plasmid	CO92	AL109969	Parkhill et al. 1999
Y. pestis plasmid pCRY plasmid	91001	AE017044	Song et al. 2004
Y. pseudotuberculosis pGDT4 plasmid		NC_011759	Lesic et al. 2012
Y .pestis	BD pool	N/A	Bos et al. 2011
Y. pestis	6330	N/A	Bos et al. 2011
Y. pestis	A120	N/A	Wagner et al. 2014

Table S2. Assembly comparison between published assemblies and SPAdes assemblies used in the current

Isolate	Velvet #Contigs	Velvet Assembly Size	SPAdes #Contigs	SPAdes Assembly Size
42013	877	4495101	303	4568982
CMCC49003	795	4534555	262	4630120
945	1243	4226749	429	4375697
164	1053	4444813	341	4552717
CMCC8211	797	4417626	249	4504619
42095	1119	4512584	324	4590055
CMCC42007	1040	4440824	340	4554624
CMCC43032	1049	4512744	336	4556201
2330	798	4427639	243	4517153
CMCC38001	1030	4483258	308	4590869
A1956001	852	4508534	254	4591409
42082	2049	4496670	365	4519613
CMCC21106	1387	4251990	434	4399440
42091	937	4495831	280	4598652
G8786	829	4325798	269	4416332
12	1092	4368767	347	4495200
9	1336	4452201	314	4550611
CMCCN010025	1016	4437913	301	4538703
M0000002	1135	4441674	327	4540628
CMCC18019	1478	4495992	317	4564746
CMCC93014	993	4507171	183	4596487
CMCC91090	987	4418766	277	4550321
CMCC05009	1501	4501730	302	4629090
620024	1131	4503786	305	4543984
CMCC11001	1100	4423348	369	4547833
780441	817	4525764	275	4616833
K21985002	1264	4399142	311	4503236
CMCC640047	1490	4305752	456	4476514
30017	747	4591472	237	4680337
CMCC31004	1229	4413744	421	4557233
C1975003	1452	4365481	462	4520607
C1989001	934	4588735	232	4687092
710317	1119	4449879	313	4565717
CMCC05013	1360	4395097	484	4454544
5	1008	4478071	329	4526762
CMCC10012	1001	4435712	353	4539951
CMCC27002	1451	4282597	448	4442887
970754	1104	4348631	385	4484656
D1991004	1270	4506081	287	4575064
D1964002	1555	4431847	271	4578802
CMCC02041	1281	4489186	301	4580519
CMCC03001	1082	4366616	297	4470946
D1982001	896	4410882	201	4506283
D1964001	1012	4389464	244	4496795
F1954001	1058	4521000	189	4620114
E1979001	75	4847813	228	4611043
CMCC84038	2446	4438544	261	4597121
YN1683	1897	4302115	432	4486804
YN472	1105	4525667	214	4630961
YN1065	1395	4472301	304	4602807
E1977001	1183	4385578	402	4517691
CMCC84033	1055	4459052	265	4577128
CMCC84046	1137	4517175	216	4622644
CMCC114001	1664	4231517	482	4436935
F1946001	1383	4463713	312	4605852
YN2179	1605	4307368	488	4483024
CMCCK110001	1385	4305250	480	4458198
YN2551	880	4524315	384	4577148
YN2588	1301	4307940	454	4454563
CMCC87001	870	4510364	280	4605449
F1984001	1467	4326553	488	4476049
YN663	1285	4203251	497	4416055
CMCCK100001	1406	4520971	255	4629229
EV76	1172	4274282	418	4396959
34008	1074	4375742	373	4509366
34202	1237	4504947	309	4623849
2	1057	4453910	363	4581507
351001	1332	4433848	363	4576259
CMCC347001	1207	4514951	250	4646579
G1996006	842	4542684	259	4653305
G1996010	772	4552418	248	4649986
CMCC348002	846	4571405	258	4662187
CMCC92010	1216	4384955	311	4483454
CMCC95001	1361	4337306	402	4486521
CMCC96001	907	4517589	246	4623583
CMCC96007	1563	4303407	432	4485527
CMCC67001	1099	4340671	346	4451820
CMCC104003	1387	4494768	293	4603432
CMCC51020	1118	4420992	372	4547443
CMCC106002	1176	4424589	319	4574720
CMCC64001	955	4360492	330	4479645
H1959004	946	4470270	298	4578285
5761	945	4262011	315	4384437
735	882	4423715	223	4533373
2506	820	4488493	238	4583349
2654	2718	4352602	364	4551644
2504	975	4475655	326	4527586
I160001	1222	4399336	307	4504324
91	906	4366271	286	4481363
K11973002	73	4720694	324	4480795
A1973001	1539	4413119	313	4508985
7338	1074	4344510	263	4422519
J1963002	1119	4434765	338	4556873
CMCC125002	790	4518010	333	4582372
I1969003	1213	4512122	282	4596400
J1978002	1098	4532297	220	4630911
H1958004	1339	4195425	438	4367581
I1970005	1199	4429836	361	4556662
CMCC99103	1262	4409843	378	4588460
CMCC90027	1102	4358986	348	4483965
CMCC92004	1461	4264825	368	4437164
I2001001	1526	4509469	187	4639195
CMCC12003	1045	4341435	343	4477836
I1994006	1247	4498336	325	4598812
SHAN11	1173	4384465	358	4526436
SHAN12	1362	4371391	393	4549993
I1991001	1191	4335804	400	4464725
CMCC107004	2814	4455583	200	4639351
7	768	4539616	310	4609682
CMCC71001	1744	4254189	460	4473709
C1976001	979	4546693	198	4646430
71021	1489	4493411	349	4610941
MGJZ6	1016	4368072	372	4484396
MGJZ7	1325	4365909	381	4506007
MGJZ9	1082	4474930	340	4599363
MGJZ11	859	4516720	266	4606493
MGJZ3	1187	4453871	187	4575827
MGJZ12	1203	4438965	353	4577245

Table S3. Accession information for genes screened in the current study

Gene/Region	Locus Tag(s)/Accession	Reference
**YTB specific genes**		
ORF2	YPTB1058	Pouillot et al. 2008
ORF3	YPTB1495	Pouillot et al. 2008
ORF4	YPTB3368	Pouillot et al. 2008
R1	YPTB0872-YPTB0878	Pouillot et al. 2008
R3	YPTB2193-YPTB2201	Pouillot et al. 2008
**YP specific genes**		
filamentous phage (YpfΦ)	YPO2271-2281	Derbise et al. 2007
pla	YPPCP1.07	Sodeinde et al. 1992
YPO0387	YPO0387	Chain et al. 2004
YPO0388	YPO0388	Chain et al. 2004
YPO0389	YPO0389	Chain et al. 2004
Y3794	Y3794	Chain et al. 2004
YPO0391	YPO0391	Chain et al. 2004
YPO0392	YPO0392	Chain et al. 2004
YPO0393	YPO0393	Chain et al. 2004
YPO0394	YPO0394	Chain et al. 2004
YPO0396	YPO0396	Chain et al. 2004
YPO0397	YPO0397	Chain et al. 2004
YPO1668	YPO1668	Chain et al. 2004
YPO1670	YPO1670	Chain et al. 2004
YPO1671	YPO1671	Chain et al. 2004
YPO2084	YPO2084	Chain et al. 2004
YPO2087	YPO2087	Chain et al. 2004
YPO2088	YPO2088	Chain et al. 2004
YPO2090	YPO2090	Chain et al. 2004
YPO2091	YPO2091	Chain et al. 2004
YPO2092	YPO2092	Chain et al. 2004
YPO2093	YPO2093	Chain et al. 2004
YPO2094	YPO2094	Chain et al. 2004
YPO2095	YPO2095	Chain et al. 2004
YPO2097	YPO2097	Chain et al. 2004
YPO2100	YPO2100	Chain et al. 2004
YPO2102	YPO2102	Chain et al. 2004
YPO2103	YPO2103	Chain et al. 2004
YPO2114	YPO2114	Chain et al. 2004
YPO2261	YPO2261	Chain et al. 2004
YPO2485	YPO2485	Chain et al. 2004
YPO2486	YPO2486	Chain et al. 2004
YPO3609	YPO3609	Chain et al. 2004
YPO3610	YPO3610	Chain et al. 2004
DFR5	AF333808-AF333810	Radnedge et al. 2002
**Shared between YP and YTB**		
PDE2	YPTB1308 and y2909	Sun et al. 2014
PDE3	YPTB3308 and y3389	Sun et al. 2014
*rcs*A	YP02449 and y1741	Sun et al. 2014
htrB (ipid A acyltransferase gene)	YPTB2490	Chain et al. 2004
High pathogenicity island (HPI)/pgm	AL031866	Schubert et al. 1998
hmsHFRS operon	U22837	Lillard et al. 1997
srfA	YPTB2212	Chain et al. 2004
srfB	YPTB2213	Chain et al. 2004
9 hemolysin genes	YPTB3450–YPTB3459	Chain et al. 2004
DFR4	AF426171	Radnedge et al. 2002
DFR1	AF333796-AF333797;AF334808	Radnedge et al. 2002
DFR2	AF333798-AF333801	Radnedge et al. 2002
DFR3	AF333802-AF333804	Radnedge et al. 2002
DFR6	AF333811-AF333813	Radnedge et al. 2002
putative two-component response regulator	YE21202-19481(3154436:3155068 reverse)	Reuter et al. 2013
putative sensor protein	YE21202-19482(3155126:3157906 reverse)	Reuter et al. 2013
orf00512 putative pathogenicity island effector protein	YE21202-19483(3158094:3158528 forward)	Reuter et al. 2013
orf00513 putative type III secretion protein	YE21202-19484(3158525:3160006 forward)	Reuter et al. 2013
orf00514 putative type III secretion protein	YE21202-19485(3160008:3161219 forward)	Reuter et al. 2013
orf00515 putative type III secretion protein	YE21202-19491(3161237:3161452 forward)	Reuter et al. 2013
orf00516 hypothetical protein	YE21202-19501(3161563:3161838 forward)	Reuter et al. 2013
orf00517 putative pathogenicity island effector protein	YE21202-19511(3161893:3162474 forward)	Reuter et al. 2013
orf00518 putative type III secretion system chaperone protein	YE21202-19521(3162502:3162960 forward)	Reuter et al. 2013
orf00519 putative pathogenicity island effector protein	YE21202-19531(3162962:3164422 forward)	Reuter et al. 2013
putative pathogenicity island effector protein	YE21202-19541(3164505:3165023 forward)	Reuter et al. 2013
orf00522 putative pathogenicity island effector protein	YE21202-19551(3165027:3165437 forward)	Reuter et al. 2013
orf00523 putative pathogenicity island protein	YE21202-19561(3165418:3165930 forward)	Reuter et al. 2013
orf00524 putative pathogenicity island effector protein	YE21202-19571(3165982:3166794 forward)	Reuter et al. 2013
orf00525 putative AraC-family regulatory protein	YE21202-19572(3166888:3167595 forward)	Reuter et al. 2013
orf00526 putative pathogenicity island protein	YE21202-19573(3167604:3167819 forward)	Reuter et al. 2013
orf00527 putative uncharacterized protein (putative type III secretion apparatus)	YE21202-19574(3167816:3168091 forward)	Reuter et al. 2013
orf00528 putative type III secretion apparatus	YE21202-19575(3168136:3168384 forward)	Reuter et al. 2013
orf00529 putative pathogenicity island lipoprotein	YE21202-19576(3168381:3169112 forward)	Reuter et al. 2013
orf00531 putative pathogenicity island protein	YE21202-19577(3169129:3169722 forward)	Reuter et al. 2013
orf00533 putative pathogenicity island protein	YE21202-19578(3169719:3170342 forward)	Reuter et al. 2013
orf00534 putative secretion system protein	YE21202-19579(3170362:3171375 forward)	Reuter et al. 2013
YE21202-19580 putative pathogenicity island protein	YE21202-19580(3171484:3171855 forward)	Reuter et al. 2013
orf00538 putative type III secretion protein	YE21202-19581(3171852:3173891 forward)	Reuter et al. 2013
orf00539 putative type III secretion ATP synthase	YE21202-19582(3173869:3175233 forward)	Reuter et al. 2013
orf00540 putative uncharacterized protein (type III secretion system apparatus protein)	YE21202-19583(3175230:3175616 forward)	Reuter et al. 2013
orf00541 conserved hypothetical protein	YE21202-19584(3175597:3175995 forward)	Reuter et al. 2013
orf00542 putative type III secretion protein	YE21202-19585(3175973:3176923 forward)	Reuter et al. 2013
orf00543 putative type III secretion protein	YE21202-19586(3177040:3177690 forward)	Reuter et al. 2013
orf00544 putative type III secretion protein	YE21202-19587(3177707:3177982 forward)	Reuter et al. 2013
orf00545 putative type III secretion protein	YE21202-19588(3177984:3178778 forward)	Reuter et al. 2013
orf00547 putative type III secretion protein	YE21202-19589(3178768:3179844 forward)	Reuter et al. 2013

Table S4. Annotation of coding regions unique to *Y. pestis*

Genbank ID	Annotation
WP_000071870	5'-nucleotidase
WP_011742534	acetyltransferase
WP_016582627	acetyltransferase
WP_043906774	aldolase
WP_000018326	aminoglycoside 3'-phosphotransferase
AIL81899	aminoglycoside adenyltransferase, partial
WP_016589769	ammonia monooxygenase
WP_001909143	ArdK protein
WP_000365431	ArsR family transcriptional regulator
WP_000983249	ATP-binding protein
WP_000085162	ATPase
WP_001280522	ATPase
EWD96542	beta-lactamase SHV-1
WP_044796618	beta-lactamase TEM, partial
WP_002224363	C-type natriuretic protein
WP_002210545	chloramphenicol acetyltransferase
WP_001239997	chromosome segregation protein ParM
EZQ51649	class I integron integrase
WP_001151304	cobyric acid synthase CobQ
WP_000479535	conjugal transfer protein TraA
WP_000595749	conjugal transfer protein TraB
WP_000637386	conjugal transfer protein TraC
WP_029498585	conjugal transfer protein TraF
WP_001326396	conjugal transfer protein TraF
WP_000128596	conjugal transfer protein TraG
WP_000534551	conjugal transfer protein TraG
WP_000256129	conjugal transfer protein TraH
WP_001337757	conjugal transfer protein TraI
WP_016674165	conjugal transfer protein TraI
WP_000794249	conjugal transfer protein TraK
WP_000805625	conjugal transfer protein TraL
WP_001256487	conjugal transfer protein TraN
WP_029498583	conjugal transfer protein TraO
WP_001909285	conjugal transfer protein TraU
WP_000793435	conjugal transfer protein TraV
WP_000621288	conjugal transfer protein TraW
WP_016589851	conjugation transfer protein
EDR41126	conserved hypothetical protein
WP_000124640	Cro/CI family transcriptional regulator
WP_002210513	DeoR family transcriptional regulator
WP_000270043	diaminopimelate decarboxylase
WP_001337754	diguanylate phosphodiesterase
WP_011264040	diguanylate phosphodiesterase
AAB03082	dihydropteroate synthase
AIC76511	dihydropteroate synthase
WP_000811656	DNA helicase
WP_000201432	DNA methyltransferase
WP_000936896	DNA methyltransferase
WP_032413577	DNA replication protein
WP_000366822	DNA topoisomerase III
WP_000651490	DNA-binding protein
WP_000706865	endonuclease
WP_032440914	endonuclease
WP_000439571	esterase
WP_000997323	exonuclease
WP_000919345	Fe3+-siderophore ABC transporter permease
WP_001102700	ferredoxin
WP_000891157	flagellar transcriptional activator
ADU64769	GCN5-related N-acetyltransferase
WP_002211730	glycoside hydrolase
EAW66125	hCG2038557, partial
WP_016257346	holin
WP_002211717	hypothetical protein
WP_002211721	hypothetical protein
WP_002209171	hypothetical protein
WP_002224265	hypothetical protein
WP_002430143	hypothetical protein
WP_011117367	hypothetical protein
WP_011264031	hypothetical protein
WP_000988732	hypothetical protein
WP_000338626	hypothetical protein
WP_000268337	hypothetical protein
WP_000122923	hypothetical protein
WP_001077335	hypothetical protein
WP_001032042	hypothetical protein
WP_000260293	hypothetical protein
WP_000184110	hypothetical protein
WP_000050848	hypothetical protein
WP_000042274	hypothetical protein
WP_001093868	hypothetical protein
WP_038220024	hypothetical protein
WP_000410925	hypothetical protein
WP_000209093	hypothetical protein
WP_000651508	hypothetical protein
WP_000340223	hypothetical protein
WP_001176699	hypothetical protein
WP_000026577	hypothetical protein
WP_000477209	hypothetical protein
WP_000793769	hypothetical protein
WP_000018872	hypothetical protein
WP_001071288	hypothetical protein
WP_000170086	hypothetical protein
WP_000739139	hypothetical protein
WP_001186917	hypothetical protein
WP_015056383	hypothetical protein
WP_015063566	hypothetical protein
WP_000709517	hypothetical protein
WP_000351984	hypothetical protein
WP_001259347	hypothetical protein
WP_001049716	hypothetical protein
WP_000509509	hypothetical protein
WP_001231463	hypothetical protein
WP_000869261	hypothetical protein
WP_000122506	hypothetical protein
WP_000228720	hypothetical protein
WP_000178856	hypothetical protein
WP_001249396	hypothetical protein
WP_031969003	hypothetical protein
WP_000505707	hypothetical protein
WP_000362482	hypothetical protein
WP_001337759	hypothetical protein
WP_000268551	hypothetical protein
WP_025887059	hypothetical protein
WP_000566600	hypothetical protein
WP_000044824	hypothetical protein
WP_001125905	hypothetical protein
WP_000703827	hypothetical protein
WP_000338945	hypothetical protein
LW57_p26	hypothetical protein
WP_002437846	hypothetical protein
WP_002210543	hypothetical protein
WP_000359512	hypothetical protein
WP_001256127	hypothetical protein
WP_000004673	hypothetical protein
WP_000988987	hypothetical protein
WP_000245298	hypothetical protein
WP_014342199	hypothetical protein
WP_000018404	hypothetical protein
WP_000277953	hypothetical protein
WP_000454742	hypothetical protein
WP_015063558	hypothetical protein
WP_000954380	hypothetical protein
WP_000062185	hypothetical protein
WP_001326176	hypothetical protein
WP_033557885	hypothetical protein
WP_000516916	hypothetical protein
WP_000333619	hypothetical protein
WP_001167032	hypothetical protein
WP_000972663	hypothetical protein
WP_000422768	hypothetical protein
WP_000591074	hypothetical protein
WP_000539392	hypothetical protein
WP_000902149	hypothetical protein
WP_000057877	hypothetical protein
WP_000805800	hypothetical protein
WP_000393785	hypothetical protein
WP_000849071	hypothetical protein
WP_040063681	hypothetical protein
WP_014342194	hypothetical protein
WP_000502622	hypothetical protein
WP_000127321	hypothetical protein
WP_000375811	hypothetical protein
WP_000101263	hypothetical protein
WP_000139717	hypothetical protein
WP_000949433	hypothetical protein
WP_000932880	hypothetical protein
WP_000243801	hypothetical protein
WP_000278322	hypothetical protein
WP_040153981	hypothetical protein
WP_011872911	hypothetical protein
WP_000787563	hypothetical protein
WP_012477588	hypothetical protein
WP_002211712	hypothetical protein
WP_011117370	hypothetical protein
WP_044819505	hypothetical protein
WP_000568107	hypothetical protein
WP_016257343	hypothetical protein
WP_016257342	hypothetical protein
WP_016257336	hypothetical protein
WP_016257034	hypothetical protein
WP_016257164	hypothetical protein
WP_032485819	hypothetical protein
WP_019841189	hypothetical protein
WP_016257340	hypothetical protein
WP_016257338	hypothetical protein
WP_016257033	hypothetical protein
WP_016261354	hypothetical protein
WP_032485830	hypothetical protein
WP_016257048	hypothetical protein
WP_016257049	hypothetical protein
WP_016257334	hypothetical protein
WP_016257161	hypothetical protein
WP_016257047	hypothetical protein
WP_002211716	hypothetical protein
WP_002214484	hypothetical protein
WP_002211715	hypothetical protein
WP_016582714	hypothetical protein
WP_002209174	hypothetical protein
WP_002215653	hypothetical protein
WP_016582820	hypothetical protein
WP_016589766	hypothetical protein
WP_016589770	hypothetical protein
AHO22799	hypothetical protein AV75_29340
EQB98214	hypothetical protein B738_26192
EFK01475	hypothetical protein HMPREF9548_03796
AHE41925	hypothetical protein KP13_32092
ERO94173	hypothetical protein L454_04959
AFJ02190	hypothetical protein Q7C_1031
EYI20322	hypothetical protein SEEH6457_01156, partial
ERF93777	hypothetical protein SEEHRA23_06235
EEO11862	hypothetical protein VCC_000128
EEO11782	hypothetical protein VCC_000178
EEO11791	hypothetical protein VCC_000187
CDL87159	hypothetical protein XCR1_840119
WP_033880368	hypothetical protein, partial
WP_032495447	hypothetical protein, partial
WP_002213827	hypothetical protein, partial
WP_016582624	hypothetical protein, partial
WP_016589409	hypothetical protein, partial
WP_002209587	integrase
EFJ86500	integrase core domain protein
WP_025471033	invasin
WP_000101568	KfrA protein
ELH92803	lactose permease
WP_000381239	membrane protein
WP_002210515	membrane protein
WP_002231149	membrane protein
WP_044075679	mercury transporter
WP_001294663	mercury transporter
WP_000082530	mercury transporter MerC
WP_015060033	MerE
WP_032282339	MerR family transcriptional regulator
WP_002209169	modification methylase
EDR32645	modification methylase EcoRII
WP_012512935	multidrug efflux protein
WP_000356489	Ner
WP_000595210	NgrC
WP_000210756	nitrite reductase
WP_000809130	nuclease, partial
WP_040146162	oxidoreductase
WP_001010738	peptidase
WP_002211729	peptidase
WP_001326179	peptidase S49
WP_011566229	peptigoglycan-binding protein LysM
WP_001337693	permease
WP_000683483	permease
WP_000464631	phage head-tail adapter protein
WP_002213570	phage tail assembly protein T, partial
WP_001187970	phosphoadenosine phosphosulfate reductase
D616_p150052	phosphoglucosamine mutase
WP_000946102	phosphohydrolase
WP_016589852	phospholipase D
EFF84395	phosphotransferase enzyme family, partial
WP_000350011	plasmid replication protein
WP_000161333	plasmid stability protein
WP_000077458	plasmid stability protein StbA
WP_001207228	plasmid stablization protein ParB
WP_000139698	protein-disulfide isomerase
WP_012221072	putative mercuric reductase
WP_000064431	pyruvate dehydrogenase
WP_001282585	recombinase
WP_016589771	RemS
WP_025989321	resolvase
WP_000904895	resolvase
WP_040126179	restriction endonuclease subunit M, partial
EDR32628	RhsD protein
WP_000542259	RNA-binding protein
WP_000842134	S-(hydroxymethyl)glutathione dehydrogenase
WP_000033700	single-stranded DNA-binding protein
EFO57254	site-specific recombinase, phage integrase family
VCD_003732	StrB
WP_016582629	structural protein P5
WP_044869904	tail protein
WP_000479153	tail protein
WP_044718019	tail protein
WP_016257341	terminase
ABX87875	terminase large subunit
WP_000113282	TetR family transcriptional regulator
AAL75564	tetracycline resistance protein
WP_000344151	thymidylate kinase
WP_009485925	TniB
WP_001337696	topoisomerase II
AFN40249	TraM
WP_001061574	transcriptional regulator
WP_001043047	transcriptional regulator
WP_000790610	transglycosylase
EDR32655	transposase
WP_043018857	transposase
WP_032491178	transposase
WP_001138065	transposase
WP_044861639	transposase
WP_002361323	transposase
EIT21339	transposase
WP_044704855	transposase, partial
ELE01527	transposon Tn21 resolvase
WP_011742533	type II restriction endonuclease
KJC06788	type IV secretion protein Rhs, partial
WP_000743450	type VI secretion protein
CH45_1649	uncharacterized protein
AAC82717	unknown
AAC82753	unknown
WP_000039318	von Willebrand factor A
WP_000334825	zinc metalloprotease

Table S5. LS-BSR matrix of 32 genes described by Chain et al. (Table S3)

	YPO0387_gi|16120353:403049-405112	YPO0388_gi|16120353:405109-406425	YPO0389_gi|16120353:406492-406722	YPO0391_gi|16120353:407449-408774	YPO0392_gi|16120353:408824-409546	YPO0393_gi|16120353:409650-410891	YPO0394_gi|16120353:c411522-411235	YPO0396_gi|16120353:412139-413551	YPO0397_gi|16120353:413548-414702	YPO1668_gi|16120353:c1900097-1898862	YPO1670_gi|16120353:c1901470-1900484	YPO1671_gi|16120353:1901648-1902325	YPO2084_gi|16120353:c2366264-2363019	YPO2087_gi|16120353:c2366553-2366293	YPO2088_gi|16120353:c2367497-2366853	YPO2090_gi|16120353:2368098-2368703
SRR069129	1	1	1	0.56	0.07	0	1	1	1	0.1	0.05	0.26	1	1	1	1
SRR069093	1	1	1	0.27	0.07	0.06	1	1	1	1	1	0.61	0	0	0	0
SRR069130	1	1	1	0.55	0.07	0.06	1	1	1	1	1	1	1	1	1	1
SRR069094	1	1	1	0.31	0.07	0	1	1	1	0.1	0.05	0.26	1	1	1	1
SRR069131	1	1	1	0.58	0.07	0.11	1	1	1	1	1	1	1	1	1	1
SRR069095	1	1	1	0.34	0.07	0.05	1	1	1	1	1	1	1	1	1	1
SRR069132	1	1	1	0.29	0.07	0	1	1	1	1	1	0.26	1	1	1	1
SRR069096	1	1	1	0.35	0.07	0	1	1	1	1	1	1	1	1	1	1
SRR069097	1	1	1	0.33	0.07	0	1	1	1	1	1	1	1	1	1	1
SRR069133	1	1	1	0.33	0.07	0.05	1	1	1	1	1	0.26	1	1	1	1
SRR069098	1	1	1	0.49	0.07	0	1	1	1	1	1	1	1	1	1	1
SRR069135	1	1	1	0.55	0.07	0	1	1	1	1	1	1	1	1	1	1
SRR069134	1	1	1	0.59	0.07	0	1	1	1	1	1	1	1	1	1	1
SRR069099	1	1	1	0.32	0.07	0.05	1	1	1	1	1	0.65	1	1	1	1
SRR069100	1	1	1	0.58	0.07	0.05	1	1	1	1	1	1	1	1	1	1
SRR069136	1	1	1	0.58	0.07	0	1	1	1	1	1	1	1	1	1	1
SRR069137	1	1	1	0.36	0.07	0.05	1	1	1	1	1	1	1	1	1	1
SRR069101	1	1	1	0.56	0.07	0.08	1	1	1	1	1	0.72	1	1	1	1
SRR069138	1	1	1	0.34	0.07	0	1	1	1	1	1	1	1	1	1	1
SRR069102	1	1	1	0.56	0.07	0.13	1	1	1	1	1	1	1	1	1	1
SRR069139	1	1	1	0.56	0.07	0.06	1	1	1	1	1	1	1	1	1	1
SRR069103	1	1	1	1	1	1	1	1	1	1	1	1	1	1	1	1
SRR069140	1	1	1	0.47	0.07	0	1	1	1	1	1	1	1	1	1	1
SRR069104	1	1	1	0.28	0.07	0.04	1	1	1	1	1	0.26	1	1	1	1
SRR069105	1	1	1	0.58	0.07	0.06	1	1	1	1	1	1	1	1	1	1
SRR069145	1	1	1	0.31	0.07	0	1	1	1	1	1	0.63	1	1	1	1
SRR069146	1	1	0.7	0.59	0.07	0	1	1	1	1	1	1	1	1	1	1
SRR069106	1	1	1	0.36	0.07	0	1	1	1	1	1	1	1	1	1	1
SRR069148	1	1	1	0.58	0.07	0.09	1	1	1	1	1	1	1	1	1	1
SRR069107	1	1	1	0.58	0.07	0.07	1	1	1	1	1	1	1	1	1	1
SRR069108	1	1	1	0.57	0.07	0	1	1	1	1	1	1	1	1	1	1
SRR069149	1	1	1	1	1	1	1	1	1	1	1	1	1	1	1	1
SRR069151	1	1	1	0.57	0.07	0.11	1	1	1	1	1	1	1	1	1	1
SRR069109	1	1	1	0.54	0.07	0.08	1	1	1	1	1	1	1	1	1	1
SRR069110	1	1	1	0.56	0.07	0	1	1	1	1	1	1	1	1	1	1
SRR069152	1	1	1	0.6	0.07	0	1	1	1	1	1	1	1	1	1	1
SRR069153	1	1	1	0.32	0.07	0	1	1	1	1	1	0.26	1	1	1	1
SRR069111	1	1	1	0.31	0.07	0	1	1	1	1	1	0.63	1	1	1	1
SRR069154	1	1	1	1	1	1	1	1	1	1	1	1	1	1	1	1
SRR069113	1	1	1	0.57	0.07	0.07	1	1	1	1	1	1	1	1	1	1
SRR069114	1	0.86	1	0.57	0.07	0.07	1	1	1	1	1	1	1	1	1	1
SRR069155	1	1	1	0.58	0.07	0.12	1	1	1	1	1	1	1	1	1	1
SRR069115	1	1	1	0.41	0.07	0.08	1	1	1	1	1	1	1	1	1	1
SRR069156	1	1	1	0.28	0.07	0	1	1	1	1	1	0.5	1	1	1	1
SRR069116	1	1	1	0.33	0.07	0.07	1	1	1	1	1	0.62	1	1	1	1
SRR069157	1	1	1	0.27	0.07	0.05	1	1	1	1	1	0.29	1	1	1	1
SRR069158	1	1	1	0.31	0.07	0	1	1	1	1	1	0.45	1	1	1	1
SRR069117	1	1	1	0.31	0.07	0	1	1	1	1	1	1	1	1	1	1
SRR069159	1	1	1	0.58	0.07	0	1	1	1	1	1	1	1	1	1	1
SRR069118	1	1	1	0.57	0.72	0.8	1	1	1	1	1	1	1	1	1	1
SRR069160	1	1	1	0.6	0.07	0.67	1	1	1	1	1	1	1	1	1	1
SRR069119	1	1	1	1	1	1	1	1	1	1	1	1	1	1	1	1
SRR069161	1	1	1	0.58	0.07	0.06	1	1	1	1	1	1	1	1	1	1
SRR069120	1	1	1	0.57	0.07	0.07	1	1	1	1	1	1	1	1	1	1
SRR069162	1	1	1	0.57	0.07	0	1	1	1	1	1	1	1	1	1	1
SRR069121	1	1	1	0.34	0.07	0	1	1	1	1	1	1	1	1	1	1
SRR069163	1	1	1	0.43	0.07	0	1	1	1	1	1	1	1	1	1	1
SRR069122	1	1	1	0.57	0.07	0	1	1	1	1	1	1	1	1	1	1
SRR069123	1	1	1	1	1	1	1	1	1	1	1	1	1	1	1	1
SRR069164	1	1	1	0.38	0.07	0.05	1	1	1	1	1	1	1	1	1	1
SRR069165	1	1	1	0.55	0.07	0	1	1	1	1	1	1	1	1	1	1
SRR069125	1	1	1	0.58	0.07	0.12	1	1	1	1	1	1	1	1	1	1
SRR069166	1	1	1	0.57	0.07	0.08	1	1	1	1	1	1	1	1	1	1
SRR069126	1	1	1	0.66	0.07	0.09	1	1	1	1	1	1	1	1	1	1
SRR069167	1	1	1	0.58	0.07	0.08	1	1	1	1	1	1	1	1	1	1
SRR069127	1	1	1	1	1	1	1	1	1	1	1	1	1	1	1	1
SRR069168	1	1	1	0.36	0.07	0	1	1	1	1	1	1	1	1	1	1
SRR069128	1	1	1	1	0.49	0.99	1	1	1	1	1	1	1	1	1	1
SRR069141	1	1	1	1	1	1	1	1	1	1	1	1	1	1	1	1
SRR069169	1	1	1	0.31	0.07	0	1	1	1	1	1	0.26	1	1	1	1
SRR069142	1	1	1	0.32	0.07	0	1	1	1	1	1	1	1	1	1	1
SRR069170	1	1	1	0.54	0.07	0.04	1	1	1	1	1	1	1	1	1	1
SRR069143	1	1	1	0.59	0.07	0.16	1	1	1	1	1	1	1	1	1	1
SRR069171	1	1	1	0.33	0.07	0.07	1	1	1	1	1	0.26	1	1	1	1
SRR069144	1	1	1	0.32	0.07	0	1	1	1	1	1	0.26	1	1	1	1
YP_Antiqua_E	1	1	1	1	1	1	1	1	1	1	1	1	1	1	1	1
YP_Angola	1	1	1	1	0.99	0.99	1	1	1	0.52	1	0.99	1	1	1	1
SRR069172	1	1	1	0.33	0.07	0	1	1	1	1	1	1	1	1	1	1
YP_Antiqua	1	1	1	1	1	1	1	1	1	1	1	1	0	0	0	0
SRR069173	1	1	1	0.28	0.07	0	1	1	1	1	1	0.26	1	1	1	1
SRR069174	1	1	1	0.3	0.07	0	1	1	1	1	1	1	1	1	1	1
SRR069175	1	1	0.7	0.33	0.07	0	1	1	1	1	1	0.26	1	1	1	1
SRR069176	1	1	1	0.58	0.07	0.1	1	1	1	1	1	1	1	1	1	1
SRR069177	1	1	1	0.34	0.07	0	1	1	1	1	1	1	1	1	1	1
SRR069178	1	1	1	0.57	0.07	0	1	1	1	1	1	1	1	1	1	1
SRR069180	1	1	1	0.57	0.07	0.07	1	1	1	1	1	1	1	1	1	1
SRR069181	1	1	1	1	1	1	1	1	1	1	1	1	1	1	1	1
SRR069182	1	1	1	0.55	0.07	0.41	1	1	1	1	1	1	1	1	1	1
SRR069183	1	1	1	0.57	0.07	0	1	1	1	1	1	1	1	1	1	1
SRR069184	1	1	1	0.37	0.07	0.07	1	1	1	1	1	1	1	1	1	1
SRR069185	1	1	1	0.43	0.07	0.1	1	0.98	1	1	1	1	1	1	1	1
SRR069186	1	1	1	0.56	0.07	0	1	1	1	1	1	1	1	1	1	1
SRR069187	1	1	1	0.99	0.69	1	1	1	1	1	1	1	1	1	1	1
SRR069188	1	1	1	0.33	0.07	0.06	1	1	1	1	1	0.26	1	1	1	1
SRR069189	1	1	1	0.56	0.07	0.08	1	1	1	1	1	1	1	1	1	1
SRR069190	1	1	1	0.38	0.07	0	1	0.99	1	1	1	0.86	1	1	1	1
SRR069191	1	1	1	0.32	0.07	0	1	1	1	1	1	1	1	1	1	1
SRR069192	1	1	1	0.31	0.07	0.59	1	0.98	1	1	1	1	1	1	1	1
SRR069193	1	1	1	1	1	1	1	1	1	1	1	1	1	1	1	1
SRR069194	1	1	1	0.28	0.07	0	1	1	1	1	1	0.37	1	1	1	1
SRR069195	1	1	1	0.34	0.07	0	1	1	1	1	1	1	1	1	1	1
SRR069196	1	1	1	0.32	0.07	0	1	1	1	1	1	1	1	1	1	1
SRR069197	1	1	1	0.33	0.07	0.06	1	1	1	1	1	0.59	1	1	1	1
SRR069198	1	1	0.7	0.59	0.07	0	1	0.99	1	1	1	1	1	1	1	1
SRR069199	1	1	1	0.57	0.07	0.06	1	1	1	1	1	1	1	1	1	1
SRR069200	1	1	1	0.56	0.07	0.08	1	1	1	1	1	1	1	1	1	1
SRR069201	1	1	1	0.33	0.07	0	1	1	1	1	1	1	1	1	1	1
SRR069202	1	1	1	1	1	1	1	1	1	1	1	1	1	1	1	1
SRR069203	1	1	1	0.31	0.07	0	1	1	1	1	1	1	1	1	1	1
SRR069204	1	1	1	0.55	0.07	0	1	1	1	1	1	1	1	1	1	1
SRR069205	1	1	1	0.34	0.07	0	1	1	1	1	1	1	1	1	1	1
SRR069206	1	1	1	0.43	0.07	0	1	0.98	1	1	1	1	0	0	0	0
SRR069207	1	1	1	0.44	0.07	0.53	1	0.98	1	1	1	1	0	0	0	0
SRR069208	1	1	1	0.57	0.07	0.06	1	1	1	1	1	1	1	1	1	1
SRR069209	1	1	0.7	0.33	0.07	0.06	1	1	1	1	1	1	1	1	1	1
SRR069210	1	1	1	0.28	0.07	0.06	1	1	1	1	1	0.26	1	1	1	1
SRR069212	1	1	1	0.54	0.07	0.07	1	1	1	1	1	1	1	1	1	1
SRR069213	1	1	0.7	0.32	0.07	0	1	1	1	1	1	0.35	1	1	1	1
SRR069214	1	1	1	1	1	1	1	1	1	1	1	1	1	1	1	1
SRR069215	1	1	1	0.31	0.07	0.05	1	1	1	1	1	0.26	1	1	1	1
SRR069216	1	1	1	0.64	1	1	1	1	1	1	1	1	1	1	1	1
YP_Antiqua_U	1	1	1	1	1	1	1	1	1	1	1	1	0	0	0	0
YP_CA88_41	1	1	1	1	1	1	1	1	1	1	1	1	1	1	1	1
YP_CO92	1	1	1	1	1	1	1	1	1	1	1	1	1	1	1	1
YP_Kim10	1	1	1	1	1	1	1	1	1	1	1	1	1	1	1	1
YP_Microtus_	1	1	1	0.99	0.99	1	1	1	1	1	1	1	1	1	1	1
YP_Nepal516	1	1	1	1	1	1	1	1	1	1	1	1	1	1	1	1
YP_Orientalis_	1	1	1	1	1	1	1	1	1	1	1	1	1	1	1	1
YP_Orientalis_	1	1	1	1	1	1	1	1	1	1	1	1	1	1	1	1
YP_Orientalis_	1	1	1	1	1	1	1	1	1	1	1	1	1	1	1	1
YP_Pestoides	1	1	1	1	0.99	1	1	1	1	1	1	1	1	1	1	1
YP_india_195	1	1	1	1	1	1	1	1	1	1	1	1	1	1	1	1
YP_pestoides_	1	1	1	0.99	0.99	1	1	1	0.5	1	1	1	1	1	1	1

	YPO2091_gi|16120353:2368704-2369093	YPO2092_gi|16120353:2369097-2369315	YPO2093_gi|16120353:2369364-2369927	YPO2094_gi|16120353:c2370194-2370012	YPO2095_gi|16120353:2370351-2370560	YPO2097_gi|16120353:2370955-2371275	YPO2100_gi|16120353:2372723-2373520	YPO2102_gi|16120353:2374643-2375092	YPO2103_gi|16120353:2375102-2377915	YPO2114_gi|16120353:2383665-2384249	YPO2261_gi|16120353:2541334-2541933	YPO2485_gi|16120353:c2786409-2786212	YPO2486_gi|16120353:c2788452-2786419	YPO3609_gi|16120353:4023133-4024503	YPO3610_gi|16120353:4024505-4024990	y3794_gi|22123922:c4213821-4213154
SRR069129	1	1	1	1	1	1	1	1	1	1	1	1	0.42	1	1	1
SRR069093	0.35	0	0	0	0	0	0	0	0	0	1	0.88	0.42	1	1	1
SRR069130	1	1	1	1	1	1	1	1	1	1	1	1	0.42	1	1	1
SRR069094	1	1	1	1	1	1	1	1	1	1	1	1	0.42	1	1	1
SRR069131	1	1	1	1	1	1	1	1	1	1	1	1	0.42	1	1	1
SRR069095	1	1	1	1	1	1	1	1	1	1	1	1	0.42	1	1	1
SRR069132	1	1	1	1	1	1	1	1	0.97	1	0.57	0.53	0.42	1	0.78	1
SRR069096	1	1	1	1	1	1	1	1	0.97	1	1	1	0.42	1	1	1
SRR069097	1	1	1	1	1	1	1	1	1	1	1	1	0.52	1	1	1
SRR069133	1	1	1	1	1	1	1	1	1	1	1	0.22	0.54	1	1	1
SRR069098	1	1	1	1	1	1	1	1	1	1	1	1	0.52	1	1	1
SRR069135	1	1	1	1	1	1	1	1	1	1	1	1	0.52	1	1	1
SRR069134	1	1	1	1	1	1	1	1	1	1	1	1	0.4	1	1	1
SRR069099	1	1	1	1	1	1	1	1	1	1	1	1	0.49	1	1	1
SRR069100	1	1	1	1	1	1	1	1	1	1	1	1	0.42	1	1	1
SRR069136	1	0.56	1	1	1	1	1	1	0.97	1	1	1	0.42	1	1	1
SRR069137	1	1	1	1	1	1	1	1	1	1	1	1	0.47	1	1	1
SRR069101	1	1	1	1	1	1	1	1	1	1	1	1	1	1	1	0.69
SRR069138	1	1	1	1	1	1	1	1	1	1	1	1	1	1	1	1
SRR069102	1	1	1	1	1	1	1	1	1	1	1	1	0.42	1	1	1
SRR069139	1	1	1	1	1	1	1	1	1	1	1	1	0.52	1	1	1
SRR069103	1	1	1	1	1	1	1	1	1	1	1	1	0.43	1	1	1
SRR069140	1	1	1	1	1	1	1	1	1	1	1	1	0.59	1	1	1
SRR069104	1	1	1	1	1	1	1	1	1	1	1	0.83	0.52	1	1	1
SRR069105	1	1	1	1	1	1	1	1	1	1	1	1	0.42	1	1	1
SRR069145	1	1	1	1	1	1	1	1	1	1	1	1	0.42	1	1	1
SRR069146	1	1	1	1	1	1	1	1	1	1	1	1	0.49	1	1	1
SRR069106	1	1	1	1	1	1	1	1	1	1	1	1	0.49	1	1	1
SRR069148	1	1	1	1	1	1	1	1	1	1	1	1	0.48	1	1	1
SRR069107	1	1	1	1	1	1	1	1	1	1	1	1	0.42	1	1	1
SRR069108	1	1	1	1	1	1	1	1	1	1	1	1	0.42	1	1	1
SRR069149	1	1	1	1	1	1	1	1	1	1	1	1	0.62	1	1	1
SRR069151	1	1	1	1	1	1	1	1	1	1	1	1	0.5	1	1	1
SRR069109	1	1	1	1	1	1	1	1	1	1	1	1	0.52	1	1	1
SRR069110	1	1	1	1	1	1	1	1	1	1	1	1	0.42	1	1	1
SRR069152	1	1	1	1	1	1	1	1	1	1	1	1	0.42	1	1	1
SRR069153	1	1	1	1	1	1	1	1	1	1	1	0.58	0.52	1	1	1
SRR069111	1	1	1	1	1	1	1	1	1	1	1	1	0.41	1	1	1
SRR069154	1	1	1	1	1	1	1	1	1	1	1	1	0.42	1	1	1
SRR069113	1	1	1	1	1	1	1	1	1	1	1	1	0.6	1	1	1
SRR069114	1	1	1	1	1	1	1	1	0.97	1	1	1	0.61	1	1	1
SRR069155	1	1	1	1	0.68	0	0	0	0	0	1	1	0.99	1	1	1
SRR069115	1	1	1	1	1	1	1	1	1	1	1	1	0.42	1	1	1
SRR069156	1	1	1	1	1	1	1	1	1	1	1	1	0.42	1	1	1
SRR069116	1	1	1	1	1	1	1	1	1	1	1	1	0.61	1	1	1
SRR069157	1	1	1	1	1	1	1	1	1	1	0.64	0.34	0.54	1	1	1
SRR069158	1	1	1	1	1	1	1	1	1	1	1	0.22	0.42	1	1	1
SRR069117	1	0.56	1	1	1	1	1	1	0.97	1	1	1	0.61	1	1	1
SRR069159	1	1	1	1	1	1	1	1	1	1	1	1	0.41	1	1	1
SRR069118	1	1	1	1	1	1	1	1	1	1	1	1	0.42	1	1	1
SRR069160	1	1	1	1	1	1	1	1	1	1	1	1	0.42	1	1	1
SRR069119	1	1	1	1	1	1	1	1	1	1	1	1	0.42	1	1	1
SRR069161	1	1	1	1	1	1	1	1	1	1	1	1	0.42	1	1	1
SRR069120	1	1	1	1	1	1	1	1	1	1	1	1	0.42	1	1	1
SRR069162	1	1	1	1	1	1	1	1	1	1	1	1	1	1	1	1
SRR069121	1	1	1	1	1	1	1	1	1	1	1	1	1	1	1	1
SRR069163	1	1	1	1	1	1	1	1	0.97	1	1	1	0.6	1	1	1
SRR069122	1	1	1	1	1	1	1	1	1	1	1	1	0.42	1	1	1
SRR069123	1	1	1	1	1	1	1	1	1	1	1	1	0.6	1	1	1
SRR069164	1	1	1	1	1	1	1	1	0.97	1	1	1	0.48	1	1	1
SRR069165	1	1	1	1	1	1	1	1	1	1	1	1	0.49	1	1	1
SRR069125	1	1	1	1	1	1	1	1	1	1	1	1	0.42	1	1	1
SRR069166	1	1	1	1	1	1	1	1	1	1	1	1	0.7	1	1	1
SRR069126	1	1	1	1	1	1	1	1	1	1	1	1	0.42	1	1	1
SRR069167	1	1	1	1	1	1	1	1	1	1	1	1	0.42	1	1	1
SRR069127	1	1	1	1	1	1	1	1	1	1	1	1	0.43	1	1	1
SRR069168	1	1	1	1	1	1	1	1	1	1	1	1	0.42	1	1	1
SRR069128	1	1	1	1	1	1	1	1	1	1	1	1	0.48	1	1	1
SRR069141	1	1	1	1	1	1	1	1	1	1	1	1	0.54	1	1	1
SRR069169	1	1	1	1	1	1	1	1	1	1	1	0.53	0.42	1	1	1
SRR069142	1	1	1	1	1	1	1	1	1	1	1	1	0.42	1	1	1
SRR069170	1	1	1	1	1	1	1	1	1	1	1	1	0.6	1	1	1
SRR069143	1	1	1	1	1	1	1	1	1	1	1	1	0.42	1	1	1
SRR069171	1	1	1	1	1	1	1	1	1	1	1	0.69	0.52	1	1	1
SRR069144	1	1	1	1	1	1	1	1	1	1	0.48	0.23	0.42	1	0.75	1
YP_Antiqua_E	1	1	1	1	1	1	1	1	1	1	1	1	1	1	1	1
YP_Angola	1	0.99	1	1	1	1	1	1	1	1	1	1	1	0.07	0	1
SRR069172	1	1	1	1	1	1	1	1	1	1	1	1	0.42	1	1	1
YP_Antiqua	0.35	0	0	0	0	0	0	0	0	0	1	1	1	1	1	1
SRR069173	1	1	1	1	1	1	1	1	1	1	0.49	0	0.59	1	1	1
SRR069174	1	1	1	1	1	1	1	1	1	1	1	1	0.52	1	1	1
SRR069175	1	1	1	1	1	1	1	1	1	1	1	0.69	0.92	1	1	1
SRR069176	1	1	1	1	1	1	1	1	0.97	1	1	1	0.52	1	1	1
SRR069177	1	1	1	1	1	1	1	1	1	1	1	1	0.47	1	1	1
SRR069178	1	1	1	1	1	1	1	1	1	1	1	1	0.42	1	1	1
SRR069180	1	1	1	1	1	1	1	1	1	1	1	1	0.48	1	1	1
SRR069181	1	1	1	1	1	1	1	1	1	1	1	1	0.42	1	1	1
SRR069182	1	1	1	1	1	1	1	1	1	1	1	1	0.52	1	1	1
SRR069183	1	1	1	1	1	1	1	1	1	1	1	1	0.61	1	1	1
SRR069184	1	1	1	1	0.68	0	0	0	0	0	1	1	0.99	1	1	1
SRR069185	1	1	1	1	1	1	1	1	1	1	1	1	0.61	1	1	1
SRR069186	1	1	1	1	1	1	1	1	0.96	1	1	1	0.42	1	1	1
SRR069187	1	1	1	1	0.68	0	0	0	0	0	1	1	0.79	1	1	1
SRR069188	1	1	1	1	1	1	1	1	0.96	1	1	0.91	0.78	1	1	1
SRR069189	1	1	1	1	1	1	1	1	1	1	1	1	0.52	1	1	1
SRR069190	1	1	1	1	1	1	1	1	1	1	1	1	0.6	1	1	1
SRR069191	1	1	1	1	1	1	1	1	1	1	1	1	0.61	1	1	1
SRR069192	1	1	1	1	1	1	1	1	1	1	1	1	0.49	1	1	1
SRR069193	1	1	1	1	1	1	1	1	1	1	1	1	0.43	1	1	1
SRR069194	1	1	1	1	1	1	1	1	1	1	1	0.46	0.42	1	1	1
SRR069195	1	1	1	1	0.68	0	0	0	0	0	1	1	0.61	1	1	1
SRR069196	1	1	1	1	0.68	0	0	0	0	0	1	1	0.99	1	1	1
SRR069197	1	1	1	1	1	1	1	1	1	1	1	0.88	0.42	1	1	1
SRR069198	1	1	1	1	0.99	1	1	1	0.97	1	0.99	1	0.42	1	1	1
SRR069199	1	1	1	1	1	1	1	1	1	1	1	1	0.42	1	1	1
SRR069200	1	1	1	1	1	1	1	1	0.61	1	1	1	0.42	1	1	1
SRR069201	1	1	1	1	1	1	1	1	1	1	1	1	0.42	1	1	1
SRR069202	1	1	1	1	1	1	1	1	1	1	1	1	0.43	1	1	1
SRR069203	1	1	1	1	1	1	1	1	1	1	1	1	0.52	1	1	1
SRR069204	1	1	1	1	1	1	1	1	1	1	1	1	0.61	1	1	1
SRR069205	1	1	1	1	1	1	1	1	0.61	1	1	1	0.52	1	1	1
SRR069206	0.35	0	0	0	0	0	0	0	0	0	1	0.87	0.49	1	1	1
SRR069207	0.35	0	0	0	0	0	0	0	0	0	1	0.91	0.5	1	1	1
SRR069208	1	1	1	1	1	1	1	1	1	1	1	1	0.42	1	1	1
SRR069209	1	1	1	1	1	1	1	1	1	1	1	1	0.42	1	1	0.21
SRR069210	1	1	1	1	1	1	1	1	1	1	1	0	0.78	1	1	1
SRR069212	1	1	1	1	1	1	1	1	1	1	1	1	0.59	1	1	1
SRR069213	1	1	1	1	1	1	1	1	1	1	1	0.4	0.42	1	1	1
SRR069214	1	1	1	1	1	1	1	1	1	1	1	1	0.61	1	1	1
SRR069215	1	1	1	1	1	1	1	1	1	1	1	0	0.59	1	1	1
SRR069216	1	1	1	1	1	1	1	1	1	1	1	1	0.61	1	1	1
YP_Antiqua_U	0.35	0	0	0	0	0	0	0	0	1	1	1	1	0.08	0	1
YP_CA88_41	1	1	1	1	1	1	1	1	1	1	1	1	1	1	1	1
YP_CO92	1	1	1	1	1	1	1	1	1	1	1	1	1	1	1	1
YP_Kim10	1	1	1	1	1	1	1	1	1	1	1	1	1	1	1	1
YP_Microtus_	1	1	1	1	0.68	0	0	0	0	0	1	1	0.99	1	1	1
YP_Nepal516	1	1	1	1	1	1	1	1	1	1	1	1	1	1	1	1
YP_Orientalis_	1	1	1	1	1	1	1	1	1	1	1	1	0.99	1	1	1
YP_Orientalis_	1	1	1	1	1	1	1	1	1	1	1	1	1	0.9	1	1
YP_Orientalis_	1	1	1	1	1	1	1	1	1	1	1	1	1	1	1	1
YP_Pestoides	1	1	1	1	0.99	1	1	1	0.97	1	0.99	1	1	1	1	1
YP_india_195	1	1	1	1	1	1	1	1	1	1	1	1	1	1	1	1
YP_pestoides_	1	1	1	1	0.89	0	0	0	0	0	1	1	0.99	1	1	1

Table S6. LS-BSR matrix of 9 hemolysin genes screened against *Y. pestis* genomes

	gi|51594359:c4098111-4097668	gi|51594359:c4098386-4098135	gi|51594359:c4098705-4098388	gi|51594359:c4099343-4098990	gi|51594359:c4101301-4099343	gi|51594359:c4101894-4101394	gi|51594359:c4105136-4101894	gi|51594359:c4108106-4105542	gi|51594359:c4108776-4108387	gi|51594359:c4109594-4108782
SRR069093	0.78	0	0	0	0.6	0	0.7	0.59	0	0.5
SRR069094	0.6	0	0	0	0.6	0	0.7	0.59	0	0.5
SRR069095	0.6	0	0	0	0.6	0	0.7	0.59	0	0.5
SRR069096	0.6	0	0	0	0.6	0	0.7	0.59	0	0.5
SRR069097	0.78	0	0	0	0.6	0	0.7	0.59	0	0.5
SRR069098	0.78	0	0	0	0.6	0	0.7	0.59	0	0.5
SRR069099	0.78	0	0	0	0.6	0	0.56	0.59	0	0.5
SRR069100	0.78	0	0	0	0.6	0	0.7	0.59	0	0.5
SRR069101	0.86	0	0	0	0.6	0	0.7	0.59	0	0.5
SRR069102	0.78	0	0	0	0.6	0	0.7	0.59	0	0.5
SRR069103	0.78	0	0	0	0.6	0	0.7	0.59	0	0.5
SRR069104	0.78	0	0	0	0.6	0	0.7	0.59	0	0.5
SRR069105	0.6	0	0	0	0.6	0	0.7	0.59	0	0.5
SRR069106	0.78	0	0	0	0.6	0	0.7	0.59	0	0.5
SRR069107	0.78	0	0	0	0.6	0	0.7	0.59	0	0.5
SRR069108	0.6	0	0	0	0.6	0	0.7	0.59	0	0.5
SRR069109	0.86	0	0	0	0.6	0	0.7	0.59	0	0.5
SRR069110	0.78	0	0	0	0.6	0	0.7	0.59	0	0.5
SRR069111	0.78	0	0	0	0.6	0	0.7	0.59	0	0.5
SRR069113	0.78	0	0	0	0.6	0	0.56	0.59	0	0.5
SRR069114	0.51	0	0	0	0.6	0	0.65	0.59	0	0.5
SRR069115	0.78	0	0	0	0.6	0	0.7	0.59	0	0.5
SRR069116	0.78	0	0	0	0.6	0	0.56	0.59	0	0.5
SRR069117	0.78	0	0	0	0.6	0	0.7	0.59	0	0.5
SRR069118	0.78	0	0	0	0.6	0	0.7	0.59	0	0.5
SRR069119	0.78	0	0	0	0.4	0	0.54	0.44	0	0.5
SRR069120	0.6	0	0	0	0.53	0	0.4	0.4	0	0.5
SRR069121	0.78	0	0	0	0.6	0	0.7	0.59	0	0.5
SRR069122	0.6	0	0	0	0.27	0	0.24	0.2	0	0.5
SRR069123	0.78	0	0	0	0.6	0	0.7	0.59	0	0.5
SRR069125	0.78	0	0	0	0.6	0	0.7	0.59	0	0.5
SRR069126	0.6	0	0	0	0.6	0	0.7	0.59	0	0.5
SRR069127	0.78	0	0	0	0.6	0	0.7	0.59	0	0.5
SRR069128	0.78	0	0	0	0.41	0	0.35	0.43	0	0.5
SRR069129	0.56	0	0	0	0.6	0	0.7	0.59	0	0.5
SRR069130	0.78	0	0	0	0.6	0	0.7	0.59	0	0.5
SRR069131	0.78	0	0	0	0.5	0	0.4	0.38	0	0.5
SRR069132	0.78	0	0	0	0.6	0	0.7	0.59	0	0.5
SRR069133	0.78	0	0	0	0.6	0	0.7	0.59	0	0.5
SRR069134	0.6	0	0	0	0.6	0	0.7	0.59	0	0.5
SRR069135	0.6	0	0	0	0.6	0	0.7	0.59	0	0.5
SRR069136	0.6	0	0	0	0.6	0	0.7	0.59	0	0.5
SRR069137	0.87	0	0	0	0.51	0	0.33	0.39	0	0.35
SRR069138	0.6	0	0	0	0.52	0	0.5	0.39	0	0.5
SRR069139	0.78	0	0	0	0.6	0	0.7	0.59	0	0.5
SRR069140	0.86	0	0	0	0.6	0	0.7	0.59	0	0.5
SRR069141	0.78	0	0	0	0.6	0	0.7	0.59	0	0.5
SRR069142	0.78	0	0	0	0.6	0	0.56	0.59	0	0.5
SRR069143	0.6	0	0	0	0.53	0	0.4	0.4	0	0.5
SRR069144	0.6	0	0	0	0.6	0	0.7	0.59	0	0.5
SRR069145	0.78	0	0	0	0.6	0	0.7	0.59	0	0.5
SRR069146	0.6	0	0	0	0.52	0	0.38	0.39	0	0.5
SRR069148	0.78	0	0	0	0.6	0	0.7	0.59	0	0.5
SRR069149	0.78	0	0	0	0.6	0	0.7	0.59	0	0.5
SRR069151	0.86	0	0	0	0.6	0	0.7	0.59	0	0.5
SRR069152	0.78	0	0	0	0.39	0	0.48	0.3	0	0.5
SRR069153	0.6	0	0	0	0.6	0	0.7	0.59	0	0.5
SRR069154	0.78	0	0	0	0.6	0	0.7	0.59	0	0.5
SRR069155	0.52	0	0	0	0.6	0	0.66	0.59	0	0.5
SRR069156	0.87	0	0	0	0.6	0	0.7	0.59	0	0.5
SRR069157	0.78	0	0	0	0.6	0	0.7	0.59	0	0.5
SRR069158	0.6	0	0	0	0.56	0	0.38	0.42	0	0.5
SRR069159	0.78	0	0	0	0.6	0	0.56	0.59	0	0.5
SRR069160	0.86	0	0	0	0.6	0	0.7	0.59	0	0.5
SRR069161	0.59	0	0	0	0.53	0	0.4	0.4	0	0.5
SRR069162	0.78	0	0	0	0.6	0	0.7	0.59	0	0.5
SRR069163	0.6	0	0	0	0.52	0	0.4	0.4	0	0.5
SRR069164	0.78	0	0	0	0.6	0	0.56	0.59	0	0.5
SRR069165	0.6	0	0	0	0.52	0	0.4	0.39	0	0.5
SRR069166	0.86	0	0	0	0.6	0	0.7	0.59	0	0.5
SRR069167	0.78	0	0	0	0.6	0	0.7	0.59	0	0.5
SRR069168	0.6	0	0	0	0.6	0	0.7	0.59	0	0.5
SRR069169	0.6	0	0	0	0.6	0	0.7	0.59	0	0.5
SRR069170	0.78	0	0	0	0.6	0	0.7	0.59	0	0.5
SRR069171	0.78	0	0	0	0.6	0	0.7	0.59	0	0.5
SRR069172	0.56	0	0	0	0.6	0	0.7	0.59	0	0.5
SRR069173	0.86	0	0	0	0.6	0	0.7	0.59	0	0.35
SRR069174	0.78	0	0	0	0.6	0	0.7	0.59	0	0.5
SRR069175	0.86	0	0	0	0.38	0	0.29	0.28	0	0.5
SRR069176	0.78	0	0	0	0.6	0	0.7	0.59	0	0.5
SRR069177	0.6	0	0	0	0.52	0	0.31	0.39	0	0.5
YP_CO92	0.78	0	0	0	0.6	0	0.7	0.59	0	0.5
SRR069178	0.6	0	0	0	0.6	0	0.7	0.59	0	0.5
SRR069180	0.78	0	0	0	0.6	0	0.7	0.59	0	0.5
SRR069181	0.78	0	0	0	0.6	0	0.7	0.59	0	0.5
SRR069182	0.78	0	0	0	0.36	0	0.29	0.27	0	0.5
SRR069183	0.86	0	0	0	0.6	0	0.56	0.59	0	0.35
SRR069184	0.6	0	0	0	0.51	0	0.49	0.39	0	0.5
SRR069185	0.78	0	0	0	0.6	0	0.56	0.59	0	0.5
SRR069186	0.6	0	0	0	0.6	0	0.7	0.59	0	0.5
SRR069187	0.78	0	0	0	0.6	0	0.46	0.58	0	0.5
SRR069188	0.78	0	0	0	0.6	0	0.7	0.59	0	0.5
SRR069189	0.78	0	0	0	0.6	0	0.7	0.59	0	0.5
SRR069190	0.6	0	0	0	0.6	0	0.7	0.59	0	0.5
SRR069191	0.78	0	0	0	0.6	0	0.7	0.59	0	0.35
SRR069192	0.78	0	0	0	0.41	0	0.59	0.44	0	0.5
SRR069193	0.78	0	0	0	0.29	0	0.3	0.22	0	0.5
SRR069194	0.78	0	0	0	0.27	0	0.24	0.2	0	0.5
SRR069195	0.78	0	0	0	0.6	0	0.7	0.59	0	0.47
SRR069196	0.6	0	0	0	0.6	0	0.7	0.59	0	0.5
SRR069197	0.6	0	0	0	0.21	0	0.39	0.26	0	0.5
SRR069198	0.78	0	0	0	0.6	0	0.7	0.59	0	0.5
SRR069199	0.6	0	0	0	0.6	0	0.7	0.59	0	0.5
SRR069200	0.78	0	0	0	0.6	0	0.7	0.59	0	0.5
SRR069201	0.6	0	0	0	0.6	0	0.7	0.59	0	0.5
SRR069202	0.78	0	0	0	0.6	0	0.56	0.59	0	0.5
SRR069203	0.71	0	0	0	0.6	0	0.7	0.59	0	0.5
SRR069204	0.6	0	0	0	0.6	0	0.56	0.59	0	0.35
SRR069205	0.6	0	0	0	0.52	0	0.4	0.39	0	0.5
SRR069206	0.78	0	0	0	0.6	0	0.7	0.59	0	0.5
SRR069207	0.78	0	0	0	0.6	0	0.56	0.59	0	0.5
SRR069208	0.6	0	0	0	0.6	0	0.7	0.59	0	0.5
SRR069209	0.86	0	0	0	0.52	0	0.4	0.39	0	0.5
SRR069210	0.78	0	0	0	0.28	0	0.3	0.21	0	0.5
SRR069212	0.6	0	0	0	0.37	0	0.29	0.31	0	0.5
YP_Angola	0.78	0	0	0	0.6	0	0.7	0.59	0	0.5
SRR069213	0.6	0	0	0	0.21	0	0.39	0.26	0	0.5
SRR069214	0.78	0	0	0	0.6	0	0.5	0.57	0	0.5
SRR069215	0.78	0	0	0	0.37	0	0.29	0.31	0	0.35
SRR069216	0.78	0	0	0	0.52	0	0.5	0.39	0	0.5
YP_Antiqua_B42003004	0.78	0	0	0	0.6	0	0.7	0.59	0	0.5
YP_Antiqua	0.78	0	0	0	0.6	0	0.7	0.59	0	0.5
YP_Antiqua_UG05_0454	0.78	0	0	0	0.6	0	0.7	0.59	0	0.5
YP_india_195	0.78	0	0	0	0.39	0	0.5	0.39	0	0.5
YP_Kim10	0.78	0	0	0	0.6	0	0.7	0.59	0	0.5
YP_Nepal516	0.6	0	0	0	0.39	0	0.5	0.39	0	0.5
YP_Orientalis_F1991016	0.78	0	0	0	0.39	0	0.5	0.39	0	0.5
YP_Orientalis_IP275	0.78	0	0	0	0.39	0	0.5	0.39	0	0.5
YP_Orientalis_MG05_102	0.78	0	0	0	0.39	0	0.5	0.39	0	0.5
YP_pestoides_A	0.78	0	0	0	0.6	0	0.7	0.59	0	0.5
YP_Pestoides_F	0.78	0	0	0	0.6	0	0.7	0.59	0	0.5
YP_CA88_4125	0.78	0	0	0	0.6	0	0.7	0.59	0	0.5
YP_Microtus_str_91001	0.78	0	0	0	0.6	0	0.7	0.59	0	0.5

Table S7. Breadth of reference coverage in ancient genomes

target	A120*	BlackDeath*	6330*
pla	100%	0%	0%
Chain 32	3.80%	100%	81%
Phage	0%	0%	0%
htrB	0%	0%	0%
hemolysin	0%	11%	11%
DFR1	67%	67%	100%
DFR2	0%	75%	75%
DFR3	33%	100%	67%
DFR4	6%	0%	6%
DFR5	0%	0%	0%
DFR6	0%	100%	100%
pgm	29%	96%	91%
PDE2	58%	99%	90%
PDE3	45%	100%	96%

*presence based on minimum depth of 3x across 80% of the reference

Table S8. Annotation of regions lost across the pMT plasmid

GenBank identifier	Annotation
WP_002211769	tail assembly
WP_002217458	hypothetical protein
WP_010981373	phage tail protein
WP_002228779	hypothetical protein
WP_042593452	host specificity protein
WP_002211773	phage tail protein
WP_002211774	tail protein
WP_002211775	tail protein
YPMT1.10c	minor tail fiber
WP_002211776	phage tail tape protein
YPMT1.12c	hypothetical protein

Figure S1. Phylogeny inferred with RAxML (Stamatakis, 2014) on a concatenation of SNPs identified with NASP (Engelthaler et al., 2014) including genomes from Y. pestis, Y. pseudotuberculosis, and Y. enterolitica (Table S1). SNPs only identified in Y. enterolitica were removed.

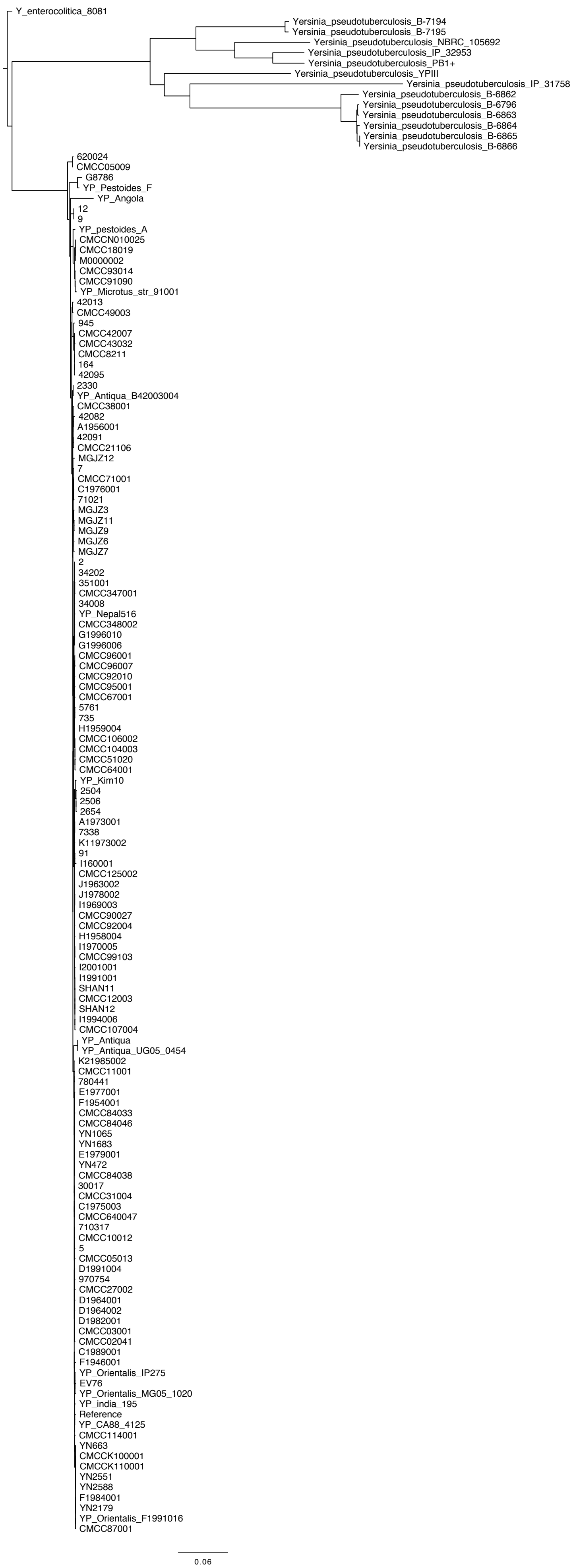

Figure S2. Distribution of coding regions (CDSs) predicted from the *pgm* locus in *Y. pestis*. Each CDS was screened against 133 *Y. pestis* genomes with LS-BSR (Sahl et al., 2014). The phylogeny was inferred with RAxML (Stamatakis, 2014) on a concatenation of SNPs identified with NASP (Engelthaler et al., 2014). The phylogeny was associated with the LS-BSR heatmap using iTOL (Letunic and Bork, 2007).

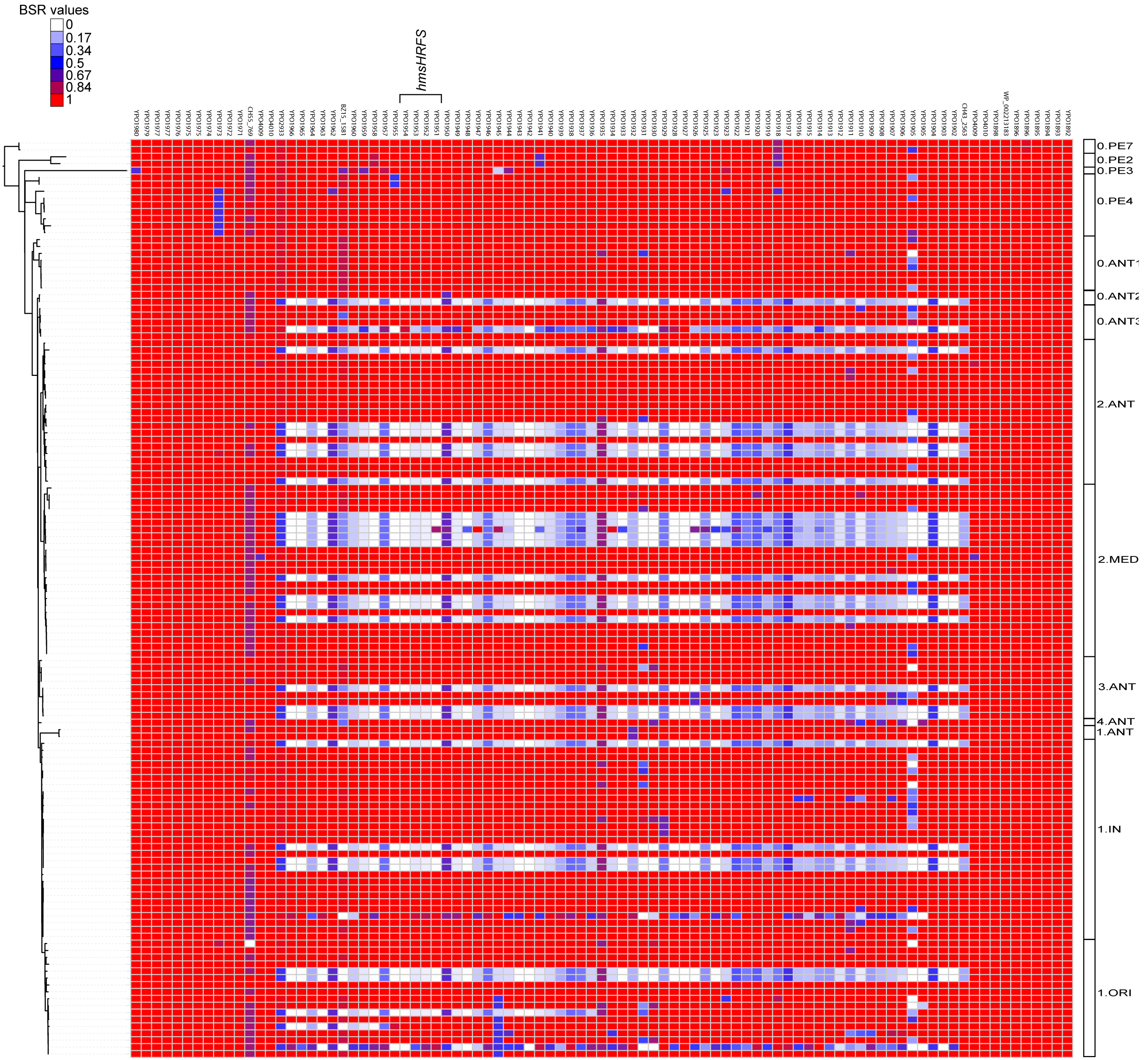

Figure S3. A section of a multiple sequence alignment (MSA) of the *srfA* gene across *Y. pestis* and *Y. pseudutuberculosis* genomes. The sequence was extracted from genomes directly from BLASTN alignments (Altschul et al., 1997), aligned with MUSCLE (Edgar, 2004), and visualized with Jalview (Waterhouse et al., 2009).

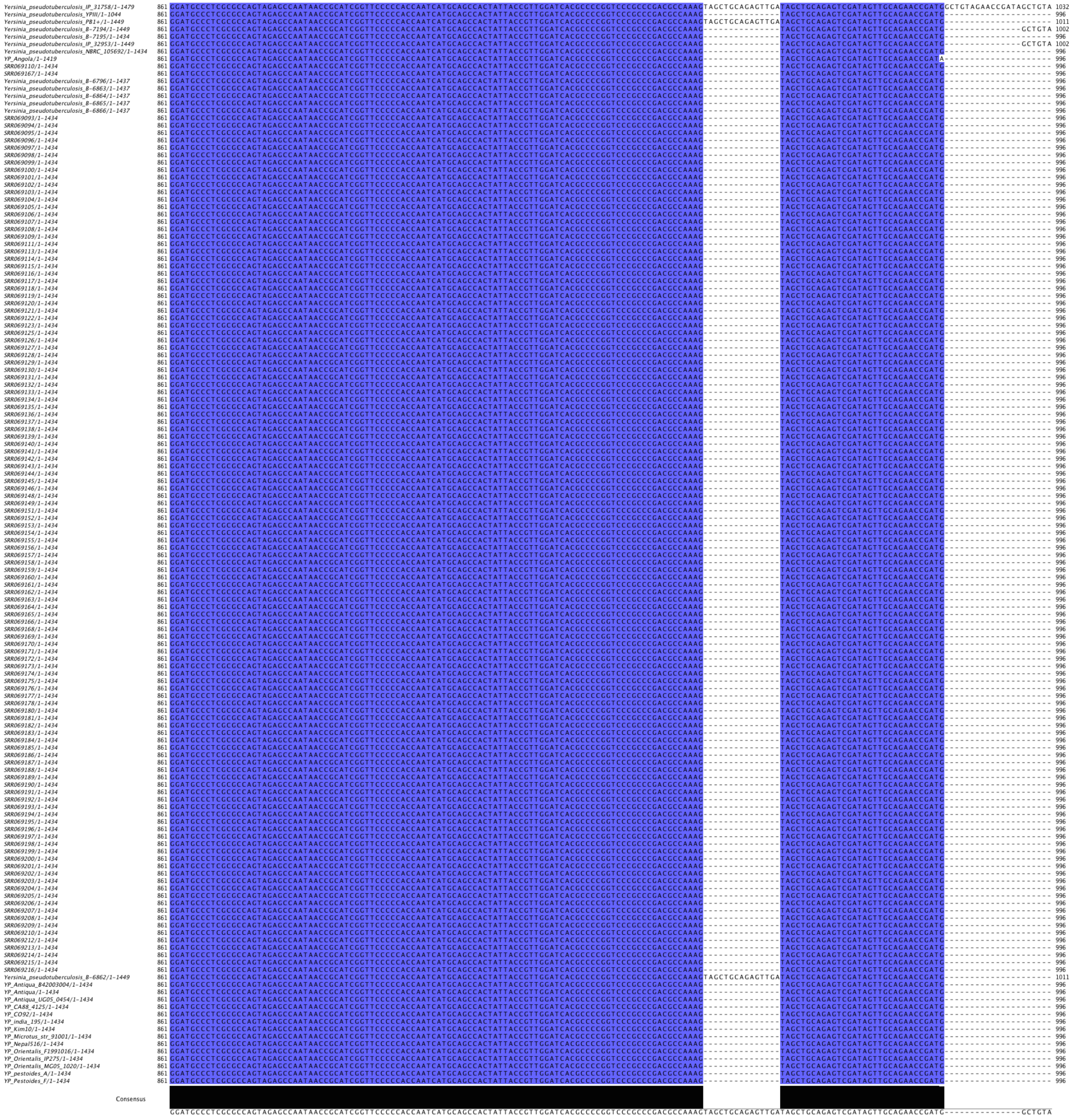

Figure S4. Distribution of genes associated with mutations in *Y. pestis* that are associated with enhanced biofilm formation and transmissibility (Sun et al., 2014). Three genes were screened against 133 *Y. pestis* genomes with LS-BSR (Sahl et al., 2014). The phylogeny was inferred with RAxML (Stamatakis, 2014) on a concatenation of SNPs identified with NASP (Engelthaler et al., 2014). The phylogeny was associated with the LS-BSR heatmap using iTOL (Letunic and Bork, 2007).

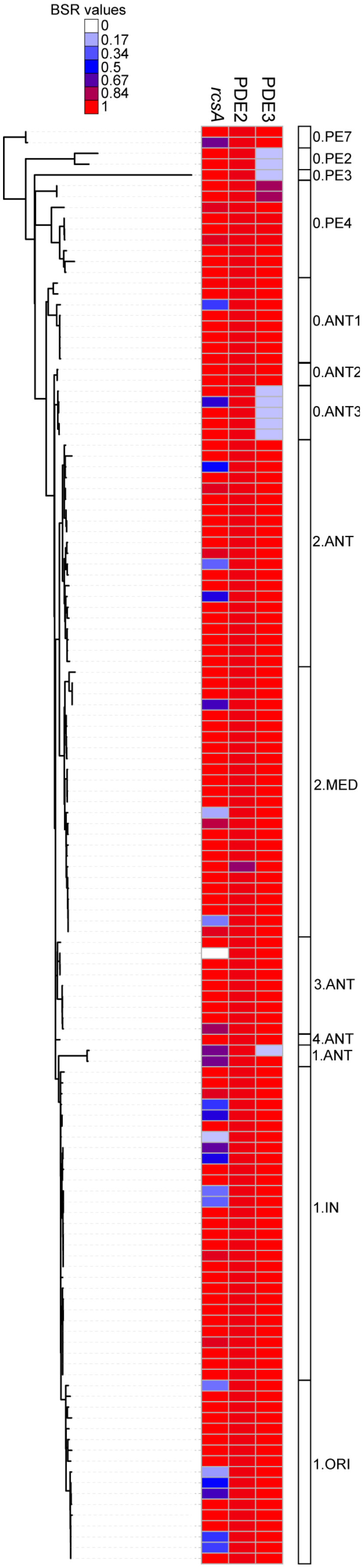

Figure S5. A section of a multiple sequence alignment (MSA) of the *rcsA* gene across *Y. pestis* genomes. The sequence was extracted from genomes from BLASTN alignments (Altschul et al., 1997), aligned with MUSCLE (Edgar, 2004), and visualized with Jalview (Waterhouse et al., 2009).

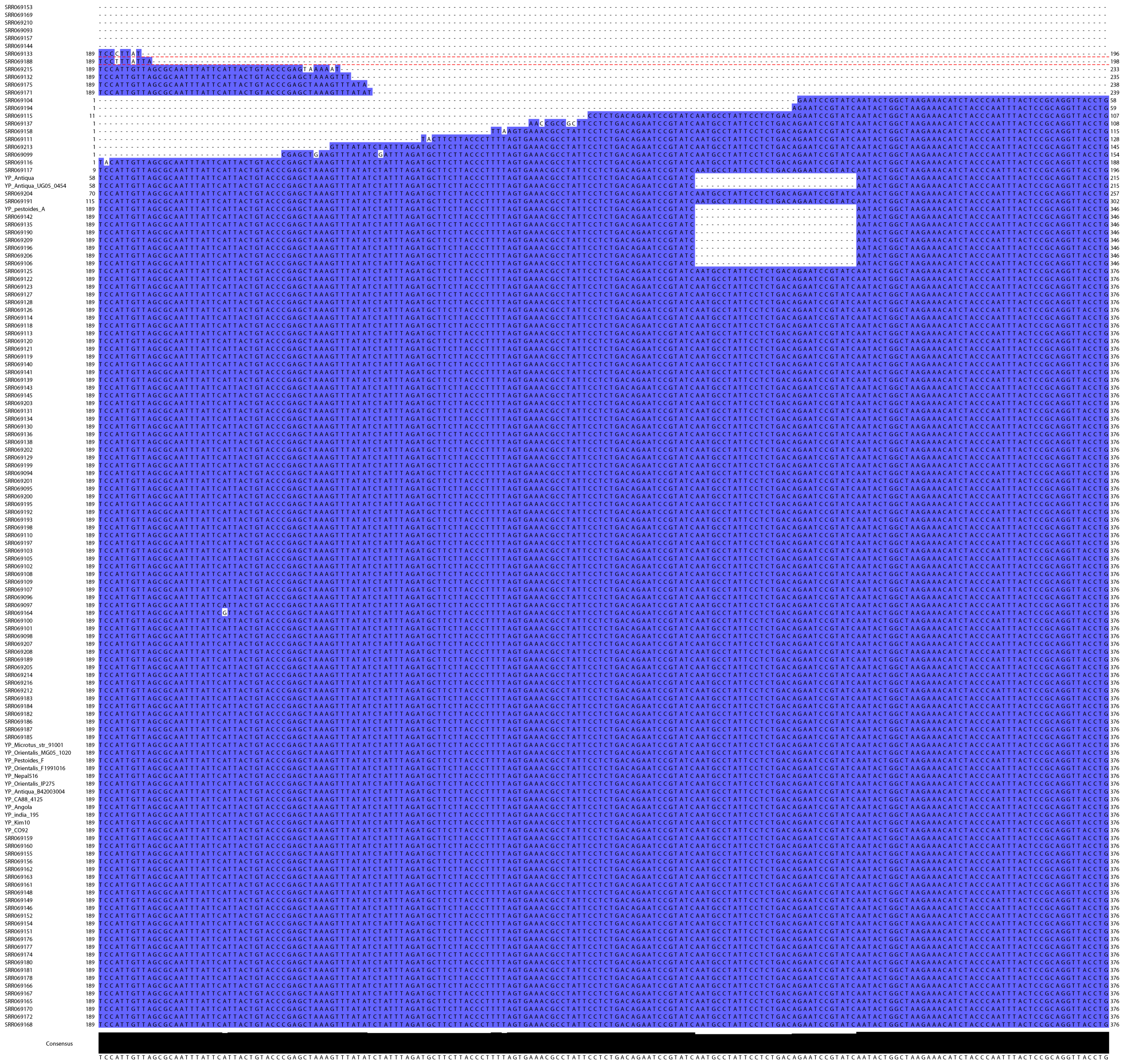

Supplement: Supplementary file 1 — Supplementary Data [file mgen-01-28-s001.pdf]
